# Comparative efficacy of various physical therapies on pain, fatigue, quality of life and functional impairment in breast cancer survivors: a network meta-analysis of randomized controlled trials

**DOI:** 10.3389/fonc.2025.1699682

**Published:** 2025-12-12

**Authors:** Yuan Luo, Qi Huang, Xiao Chen, Hongju Peng, Yu Li, Li Chen, Liyue Zhang, Yi Huang

**Affiliations:** 1 The First People’s Hospital of Neijiang City, Sichuan, Neijiang, China; 2 The Second People’s Hospital of Neijiang City, Sichuan, Neijiang, China

**Keywords:** physical therapy, manual lymphatic therapy, aerobic exercise, breast cancer, network meta-analysis

## Abstract

**Objective:**

This study aims to conduct a comparative analysis of the effects of different physical therapies on the pain, fatigue, functional impairment, quality of life, and grip strength of breast cancer survivors. Design:A systematic review and network meta-analysis were conducted.

**Methods:**

The process of screening, data extraction, coding and bias risk assessment is conducted in an independent and duplicated manner. The primary outcome measures are subjected to evaluation through the utilization of Bayesian network meta-analysis. The online Meta-analysis Confidence (CINeMA) tool is employed to assess the quality of evidence.

**The data source:**

PubMed, Cochrane Library, Web of Science and Embase.

**Eligibility criteria for selecting studies:**

This article examines any randomized controlled trials that involve physical therapy for breast cancer survivors.

**Results:**

A total of 111 RCTs involving 6888 participants and 16 types of physical therapy interventions were included. A network meta-analysis showed that all physical therapy measures had some effect on breast cancer survivors compared with placebo. Virtual reality technology may be more effective in relieving pain, electrotherapy may be more effective in restoring functional disorders, kinesiology taping may be more effective in terms of fatigue, quality of life (physical aspect), and grip strength, and aerobic exercise may be more effective in relieving Quality of life (Mental Component). The final curvature under the cumulative sequence curve indicates that virtual reality technology, intramuscular adhesives, and mixed exercises are relatively good auxiliary treatment methods. The degree of confidence varies from high to very low according to CINeMA.

**Conclusion:**

For breast cancer survivors, mental improvements are just as important as physical improvements. Researchers should pay more attention to the overall benefits and the safety and feasibility of trials. However, this conclusion still needs to be further verified by a large number of multi-center and large sample size RCT.

## Background

Breast cancer is the most prevalent form of cancer among women (1–3). Consequently, a significant volume of research is dedicated to the management of breast cancer in various settings, including diagnosis, surgery, adjuvant therapy, and metastatic treatment (4). Breast cancer survivors frequently encounter complications such as lymphedema, limited shoulder mobility, pain, fatigue, and other health issues (5–8). These sequelae collectively represent a major clinical challenge in survivorship care, as they significantly impair physical function, psychological well-being, and overall health-related quality of life. Consequently, the development of effective rehabilitation strategies is a priority within oncological clinical practice guidelines. A meta-analysis of randomized trials has demonstrated the efficacy of physical therapy in improving function in patients with early breast cancer (9). At the time, however, there was a paucity of research on complementary treatments for breast cancer, and no conclusive research evidence existed regarding the safety or actual efficacy of most physical therapy modalities for breast cancer survivors.

The utilization of diverse physical therapy modalities has undergone a gradual transition over time. Conventional decongestant therapy plays a pivotal role in the management of lymphedema in breast cancer, encompassing manual lymphatic drainage, intermittent pneumatic compression, compression bandages or pressure garments, regular functional exercise, and skin care (10–12). Subsequent studies have seen an increase in the use of alternative physical therapy modalities, including the application of intramural tape, hydrolymphatic therapy, virtual reality technology, neuromuscular promotion technology and yoga in breast cancer survivors, thus providing survivors with a choice of physical therapy interventions (13–19). This expansion of available modalities is reflected in numerous systematic reviews, which have synthesized evidence for individual interventions. However, these reviews often focus on a single therapy or a limited set of outcomes, creating a fragmented evidence base. However, the issue remains unresolved, as no study has yet demonstrated which physical therapy modality is more beneficial for breast cancer survivors. The critical gap lies in the absence of a unified, comparative analysis that ranks these diverse interventions simultaneously to inform clinical decision-making.

The objective of this study was to evaluate the effectiveness of various physical therapy interventions for breast cancer survivors, with a particular focus on pain management and quality of life. To provide a comprehensive assessment of patient-centered outcomes, we also pre-specified several secondary outcomes, including fatigue, functional disability, and grip strength, which are commonly reported in the literature and highly relevant to daily living. To the best of our knowledge, no previous study has systematically analyzed and statistically compared diverse physical therapy techniques for this population. We conducted a comprehensive literature review and performed a network meta-analysis to evaluate the relative efficacy of these interventions. Our aim was to identify optimal physical therapy approaches and provide evidence-based clinical recommendations.

## Methods

### Search strategy

This systematic review was conducted in accordance with the Preferred Reporting Items for Systematic Reviews and Meta-Analyses (PRISMA 2020) guidelines. The literature search was conducted for articles published between January 1990 and October 2025. See Appendix for a detailed search strategy. In order to obtain a more complete data report, we also conducted a search of references from relevant systematic reviews included in the study, and conducted a manual check to obtain and identify eligible gray literature. We manually screened the reference lists of all studies included in the final analysis as well as relevant systematic reviews identified during our database search to identify any potentially eligible articles that our electronic search might have missed.

### Data selection

Inclusion criteria: (a) Randomized controlled trial; (b) Study participants were breast cancer survivors aged 18 years or older; (c)studies in which patients have received some intervention related to physical therapy (any treatment related to physical exercise, manual therapy or other complementary therapies used in clinical practice) (20); (d) Outcome measures included at least one of pain assessment, fatigue assessment, functional disability assessment, quality of life, and grip strength, and relevant data were extracted before and after treatment.

Exclusion criteria: (a) literature with incomplete data, such as meetings, abstracts, letters and reviews; (b) Duplicate published studies; (c) Studies in which literature data cannot be extracted effectively; (d) Studies where the full text is not available;(e) Pilot randomized controlled trial.

### Rationale for the broad scope of interventions

We acknowledge the methodological challenge of incorporating a wide array of physical therapy modalities, which indeed differ in their application and mechanisms of action (e.g., passive device-based therapies like electrotherapy versus active, patient-engaged modalities like exercise). The decision to include this diverse set was driven by the primary research objective: to provide a comprehensive overview and generate a hierarchy of effectiveness for the most common physical therapy interventions available in clinical practice for breast cancer survivors. This approach, while introducing clinical heterogeneity, is a recognized application of network meta-analysis (NMA) aimed at answering a pragmatic clinical question. We have addressed this inherent diversity through several measures: 1) ensuring all interventions fall under the broad, pre-specified definition of physical therapy; 2) conducting a thorough evaluation of the transitivity assumption; and 3) performing sensitivity and subgroup analyses to explore the impact of different intervention types on the overall results, as detailed in the subsequent analysis sections.

### Literature screening and data extraction

The electronic database was searched independently by two researchers (YL and LC) using EndNote software to delete duplicate studies. Relevant literature titles and abstracts were then read, and literature not relevant to the study was excluded. The selection process was conducted by the two researchers, and any objections were discussed until a consensus was reached. If a consensus could not be reached, the third researcher made the final decision after group discussion. The data were then extracted and organized according to pre-established information tables, including the first author of each study, the year of publication, the country in which the study was conducted, mean/median age of the study participants, the sample size, the intervention mode, the randomization method, the treatment cycle, and the outcome evaluation.

### Literature quality evaluation

The RCTs included were assessed for methodological bias and quality according to the Cochrane Handbook for Systematic Review of Interventions. This assessment included the generation of random sequences, assignment concealment, investigator-patient blindness, blind outcome evaluation, incomplete outcome data, selective outcome reporting, and other sources of bias (21). Assessment options include: ‘Low risk,’ ‘High risk,’ or ‘Unclear risk.’ To assess the confidence of each comparison with the control group, we also used the CINeMA online assessment system, a tool designed by Cochrane to compare multiple intervention groups as an adaptation of the GRADE network meta-analysis to determine in-study bias, reporting bias, incoherence, imprecision, heterogeneity, and inconsistency (22, 23).

### Statistical analysis

Network meta-analysis of the data was performed using Stata 17.0 software (24, 25). In this study, continuous variables were employed, and weighted mean difference (WMD) statistics were combined, with 95% confidence intervals (CIs) being calculated, including VAS, QOL, fatigue and GS. When the 95%CI value of WMD was 0, the comparison was deemed to be statistically insignificant. P < 0.05 indicated significant differences, and I² value was used to test heterogeneity. When P > 0.05 and I² ≤ 50%, it indicated small differences, and a fixed benefit model was used for network meta-analysis. Conversely, when P < 0.05 and I² > 50%, a random effects model was used to further explore the source of heterogeneity, including subgroup analysis and sensitivity analysis. For each pre-specified outcome, a global network diagram is used to illustrate a direct comparison between interventions, with the size of the nodes in the graph corresponding to the number of participants receiving each treatment. Treatments receiving direct comparisons are connected with lines whose thickness is proportional to the number of tests evaluating a particular comparison. In the results section, a cumulative probability ranking plot is used to represent the ranking probability of each intervention, with SUCRA values ranging from 0 to 100%, with higher SUCRA values indicating a higher ranking of the intervention, generally reflecting a more favorable or unfavorable effect. The ranking of interventions was conducted on the basis of SUCRA values or the area under the curve, with the objective being to calculate the ranking result of the probability cumulative ranking curve of each physical therapy intervention, to draw a ranking map, and to judge the relatively best physical therapy measures. In order to assess potential publication bias, funnel plots adjusted for comparison were used. The analysis was designed to determine whether there was evidence of small sample effects or publication bias in the intervention network.

## Results

### Literature search results

**Figure 1:**
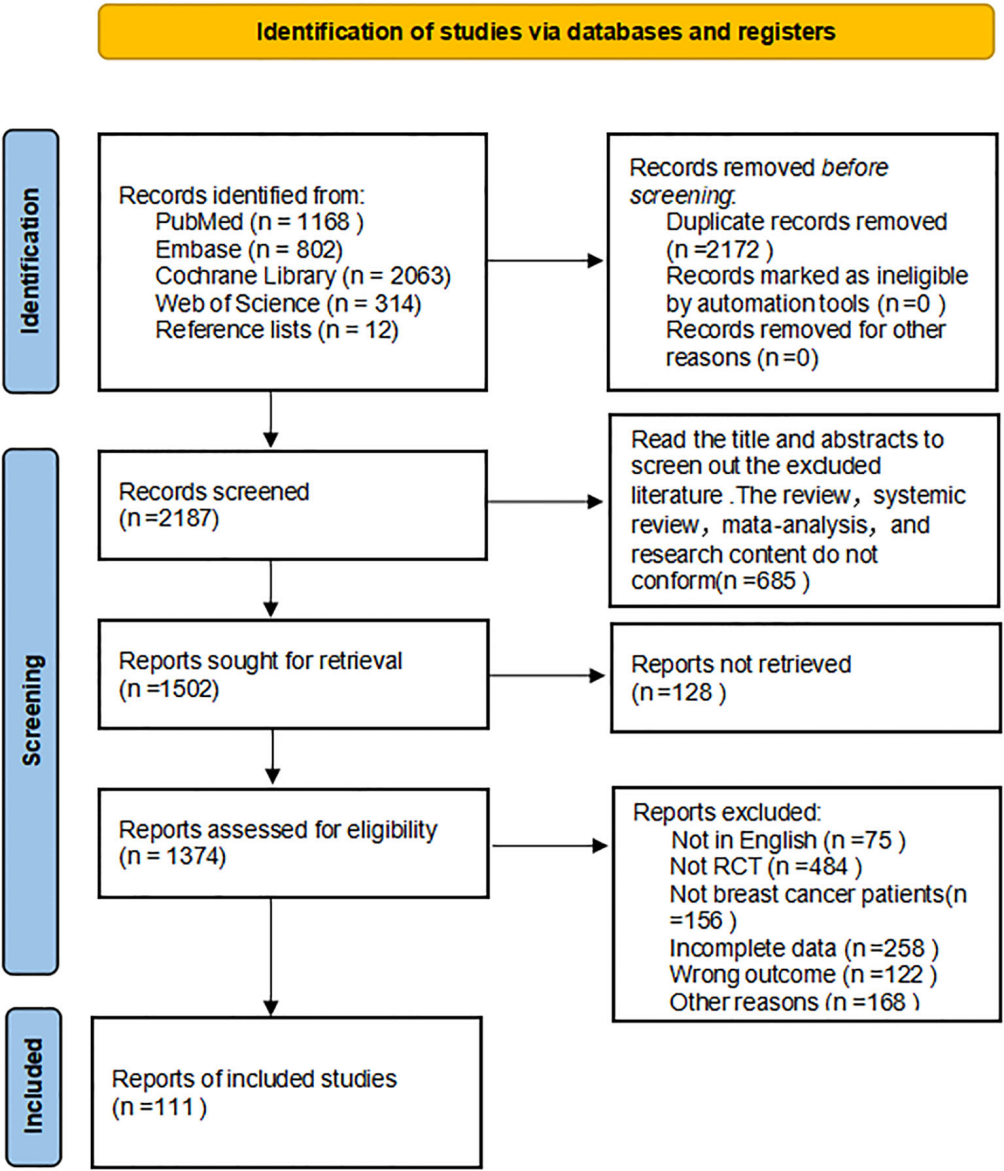
Literature screening flowchart.

As demonstrated in **Figure 1**, a total of 4,359 publications were identified (Pubmed: 1,168, Embase: 802, Cochrane Library: 2,063, Web of Science: 314, Other sources: 12) 2,172 duplicative literatures were deleted, 685 non-conforming literatures were deleted according to abstract and title, 128 were not searched reports, 1,374 literatures met full text screening, 1,263 literatures were deleted according to inclusion and exclusion criteria, and 111 (26–136) literatures were finally included. The two statisticians have a unified opinion in the process of searching and including documents.

### Basic features and quality assessment were included

A total of 111 randomized controlled trials were included in the analysis, with a total of 6,888 participants from 22 countries. The 111 studies comprised 16 distinct interventions: aquatic exercise, aerobic exercise, aqua lymphatic therapy, electrotherapy, kinesio taping, low level laser therapy, manual lymphatic drainage, mixed motion, moxibustion, pneumatic circulation, proprioceptive neuromuscular facilitation, resistance exercise, virtual reality, yoga and ultrasound therapy. The fundamental characteristics of the included studies are delineated in **Table 1**. The Cochrane systematic review of interventions described the evaluation of randomized controlled trials on seven aspects associated with the risk of bias (see **Table 2**). Please refer to **Appendix** for the CINeMA Network Meta-online evaluation.

**Table 1:** Main characteristics of the included clinical studies.

Inclusion study	Year	Intervention measure	Design	Age	N	Period	Country	Outcome
Wang	2019	MO/PC	2-arm RCT	59.42 ± 7.02 58.25 ± 6.19	24/24	4WK	China	VAS;FA
Liu	2023	MO/PC	2-arm RCT	58.45 ± 5.92 59.3 ± 7.06	20/20	4WK	China	VAS
Lampinen	2021	PB/MLD	2-arm RCT	60.34 ± 10.65 64.24 ± 13.69	15/13	4WK	America	DASH
Atef	2020	VR/PNF	2-arm RCT	54.07 ± 8.28 53.07 ± 7.24	18/18	4WK	Egypt	DASH
Haines	2010	RET/MM	2-arm RCT	55.9 ± 10.5 54.2 ± 11.5	46/43	12WK	Australia	VAS;GS
Kilbreat	2020	MM/PB	2-arm RCT	59.5 ± 8 53.7 ± 10.4	41/47	12WK	Australia	VAS
Dayes	2013	MLD/PB	2-arm RCT	61 ± 38.03 58.65 ± 26.78	57/46	3WK、6WK	Canada	DASH;QOL
Meer	2023	MLD/PB	2-arm RCT	49.84 ± 12.35 45.88 ± 11.95	19/17	4WK	Pakistan	VAS;FA;DASH
Moro	2024	AET/PB	2-arm RCT	56.4 ± 7.29 59.9 ± 9.67	18/13	12WK	Italy	QOL
Letellier	2014	ALT/PB	2-arm RCT	56.4 ± 9.76 53.4 ± 9.35	13/12	12WK	Canada	VAS;DASH;GS
Ahmed	2006	RET/PB	2-arm RCT	52.3 ± 7.7 51.7 ± 7.5	23/23	24WK	America	GS
Feyzioglu	2020	VR/PB	2-arm RCT	50.84 ± 8.53 51 ± 7.06	19/17	6WK	Turkey	VAS;DASH;GS
Baxter	2018	LLLT/PB	2-arm RCT	57.9 ± 9.6 64.3 ± 11.1	9/8	6WK、12WK	New Zealand	VAS
Ridner	2013	LLLT/MLD	2-arm RCT	66.4 ± 11.3 67.5 ± 10.3	15/16	4WK	America	FA
Ahmed	2011	LLLT/PB	2-arm RCT	54.76 ± 3.33 53.36 ± 3.56	25/25	4WK、12WK	Iran	GS
Kozanoglu	2009	PC/LLLT	2-arm RCT	51.2 ± 10.3 45.4 ± 9.9	24/23	12WK	Turkey	VAS;GS
Belmonte	2012	ET/MLD	2-arm RCT	69.56 ± 10.05 65.5 ± 12.74	18/14	4WK	Spain	VAS
Song	2020	ET/PB	2-arm RCT	49.91 ± 8.85 49.71 ± 8.24	36/36	4WK	Korea	VAS
Hemmati	2022	PB/ET/UG	3-arm RCT	49.13 ± 10.5 48.96 ± 10.12 49.32 ± 10.15	13/13/13	2WK	Iran	VAS;DASH
Robb	2007	ET/PB	2-arm RCT	–	19/15	3WK	Britain	VAS
Conejo	2018	KT/PB	2-arm RCT	67.27 ± 8.56 65.6 ± 7.23	20/20	5WK	Spain	VAS;FA;QOL
Ergin	2019	KT/PB	2-arm RCT	58.44 ± 10.12 53.42 ± 7.69	18/14	4WK	Turkey	QOL
Tantawy	2019	KT/PC	2-arm RCT	54.3 ± 4.16 55.15 ± 3.27	30/29	3WK	Egypt	VAS;FA;QOL;GS
Tsai	2009	KT/PB	2-arm RCT	–	20/21	4WK	China	QOL
Melgaard	2016	KT/PB	2-arm RCT	63 ± 9.8 62.5 ± 7.6	5/5	4WK	Denmark	QOL
Garcia	2024	MM/PB	2-arm RCT	49 ± 8.9 50.1 ± 7.9	31/28	12WK	Spain	GS
Gradalski	2015	MLD/PB	2-arm RCT	61.2 ± 9.2 62 ± 12.2	30/30	12WK	Poland	VAS
Sen	2021	MLD/PB	2-arm RCT	56 ± 13.7 57.6 ± 9.4	25/25	4WK	Turkey	VAS;DASH
Xiong	2023	MLD/PB	2-arm RCT	50.4 ± 8.8 53.5 ± 7	52/52	4WK、12WK	China	VAS
Uzkeser	2015	PC/PB	2-arm RCT	42-75 37-75	16/15	3WK	Turkey	VAS
Carrera	2024	MLD/PB	2-arm RCT	59.57 ± 10.86 60.21 ± 9.87	14/14	4WK	Spain	VAS
Oliveira	2014	MLD/MM	2-arm RCT	55.6 ± 11.9 56.7 ± 15.1	48/48	4WK	Brazil	DASH
Ergin	2017	ALT/MLD	2-arm RCT	44.5 ± 13.69 47.66 ± 16.82	30/27	6WK	Turkey	VAS;QOL
Tambour	2018	MLD/PB	2-arm RCT	62 ± 11.5 60.9 ± 10.8	38/35	4WK	Denmark	VAS
Devoogdt	2011	MLD/PB	2-arm RCT	55.8 ± 12.5 54.5 ± 11.1	77/81	12WK	Belgium	QOL
Nele	2018	MLD/PB	2-arm RCT	56 ± 13 55 ± 11	65/68	24WK	Belgium	QOL
Villanueva	2013	ALT/PB	2-arm RCT	49 ± 7 47 ± 8	32/29	8WK	Spain	FA
Ali	2021	ALT/MM	2-arm RCT	51.36 ± 9.15 49.85 ± 8.57	25/25	8WK	Egypt	VAS
McNeely	2004	MLD/PB	2-arm RCT	58 ± 13 63 ± 13	24/21	4WK	Canada	DASH
Bahtiyarca	2019	MLD/PB	2-arm RCT	55.2 ± 7.15 61.64 ± 11.69	10/14	4WK	Turkey	DASH;QOL
Conwright	2021	MM/PB	2-arm RCT	46.8 ± 10.2 55.7 ± 10.5	28/24	12WK	America	FA;QOL
Winters	2022	RET/AET	2-arm RCT	70.6 ± 5.4 71.1 ± 4.6	39/37	12WK	America	QOL
Milne	2008	MM/PB	2-arm RCT	55.2 ± 8.4 55.1 ± 8	29/29	6WK	Canada	FA
Courneya	2007	PB/RET/AET	3-arm RCT	26-78 25-76 30-75	82/82/78	12WK	Canada	FA
Sweeney	2019	MM/PB	2-arm RCT	52.8 ± 10.6 53.6 ± 10.1	50/50	16WK	Canada	DASH
Chaoul	2018	YG/AET/PB	3-arm RCT	49.5 ± 9.8 50.4 ± 10.3 49 ± 10.1	74/68/85	1WK、12WK	America	FA
Yagli	2015	YG/MM	2-arm RCT	68.58 ± 6.17 68.88 ± 2.93	10/10	4WK	Turkey	VAS;FA
Porter	2019	YG/PB	2-arm RCT	56.3 ± 11.6 59.4 ± 11.3	43/20	4WK、12WK	America	VAS;FA
Eyigor	2018	YG/PB	2-arm RCT	51.5 ± 7.3 52.3 ± 9.5	22/20	10WK	Turkey	VAS
Pasyar	2019	YG/PB	2-arm RCT	51.6 ± 10.46 51.8 ± 11.4	20/20	4WK	Iran	VAS;FA;QOL
Loudon	2016	YG/PB	2-arm RCT	55.1 ± 2.5 60.5 ± 3.6	12/11	8WK	Australia	VAS
Vadiraja	2017	YG/PB	2-arm RCT	30-70 30-70	33/31	4WK	India	FA
Bower	2012	YG/PB	2-arm RCT	54.4 ± 5.7 53.3 ± 4.9	16/15	12WK	America	FA
Banasik	2011	YG/PB	2-arm RCT	63.33 ± 6.9 62.4 ± 7.3	7/7	8WK	America	FA
Jong	2018	YG/PB	2-arm RCT	51 ± 8 51 ± 7.3	40/27	12WK	Netherland	VAS;FA
Wong	2024	YG/PB	2-arm RCT	48.63 ± 8.77 45.78 ± 9.25	16/18	4WK	China	FA;DASH;QOL
Moadel	2007	YG/PB	2-arm RCT	55.11 ± 10.07 54.23 ± 9.81	84/44	4WK、12WK	America	FA;QOL
Lotzke	2016	YG/PB	2-arm RCT	51 ± 11 51.4 ± 11.1	45/47	12WK	Germany	VAS;FA
Vadiraja	2009	YG/PB	2-arm RCT	–	44/44	6WK	India	VAS;QOL
Chandwani	2014	YG/AET/PB	3-arm RCT	52.38 ± 1.35 51.14 ± 1.32 52.11 ± 1.34	53/56/54	6WK	America	FA;QOL
Hosakote	2009	YG/PB	2-arm RCT	–	42/33	4WK	India	VAS;FA
Siedentofy	2013	YG/PB	2-arm RCT	55.82 ± 10.72 58.41 ± 9.91	31/28	5WK	Germany	QOL
Taso	2014	YG/PB	2-arm RCT	49.27 ± 10.23 49.27 ± 10.23	30/30	4WK	China	FA
Cramer	2015	YG/PB	2-arm RCT	48.3 ± 4.8 50 ± 6.7	19/21	12WK	Germany	FA;QOL
Vardar	2015	YG/AET	2-arm RCT	49.89 ± 4.65 47.38 ± 7.57	19/21	6WK	Turkey	VAS;FA
Stan	2016	RET/YG	2-arm RCT	63 ± 9.3 61.4 ± 7	16/18	6WK	America	QOL
Littman	2012	YG/PB	2-arm RCT	60.6 ± 7.1 58.2 ± 8.8	32/31	7WK	America	FA;QOL
Annette	2014	YG/PB	2-arm RCT	55.1 ± 2.5 60.5 ± 3.6	12/11	8WK	Australia	VAS;FA
Dahhak	2022	RET/PB	2-arm RCT	51 ± 5 55 ± 9	10/10	12WK	Belgium	GS
Naczk	2022	RET/PB	2-arm RCT	66.2 ± 10.6 66.2 ± 10.6	12/12	6WK	Poland	VAS
Michels	2023	AET/RET	2-arm RCT	52.38 ± 8.99 62.76 ± 9.18	24/17	3WK	Germany	QOL
Husebo	2014	MM/PB	2-arm RCT	50.8 ± 9.7 53.6 ± 8.8	33/34	18WK	Norway	FA
Buchan	2016	RET/AET	2-arm RCT	58.5 ± 10.05 53.7 ± 10.95	21/20	12WK	Australia	DASH;QOL
Singh	2016	PC/PB	2-arm RCT	52.7 ± 9.4 59.1 ± 9.8	15/24	12WK	Australia	QOL
Paulo	2019	MM/AET	2-arm RCT	63.2 ± 7.1 66.6 ± 9.6	18/18	12WK	Brazil	FA;QOL
Taradaj	2016	KT/PB	2-arm RCT	60.3 ± 4.2 63.2 ± 5.1	22/23	4WK	Poland	QOL
Liu	2022	YG/PB	2-arm RCT	51-60 51-60	68/68	8WK	China	VAS;FA
Lee	2022	RET/PB	2-arm RCT	54.7 ± 5.1 55.4 ± 4.3	15/15	12WK	Korea	GS
Cormie	2013	RET/PB	2-arm RCT	57 ± 10 58.6 ± 6.7	21/19	12WK	Australia	DASH;QOL;GS
Soidan	2020	RET/ALT/AET	3-arm RCT	65 ± 7 64 ± 6.8 66 ± 7.1	74/65/72	24WK	Spain	VAS;QOL
Bloomquist	2021	AET/PB	2-arm RCT	47.4 ± 9.4 50 ± 9.3	46/22	24WK	Denmark	DASH
Omar	2020	RET/PC	2-arm RCT	52.62 ± 2.92 53.78 ± 2.99	30/30	8WK	Egypt	VAS;DASH
Steindorf	2014	RET/PB	2-arm RCT	55.2 ± 9.6 56.4 ± 8.7	77/78	12WK	Germany	VAS;FA;QOL
Park	2023	RET/PB	2-arm RCT	58.86 ± 3.28 60.29 ± 5.09	8/8	6WK	Korea	VAS;FA;DASH;GS
Hagstrom	2016	RET/PB	2-arm RCT	51.2 ± 8.5 52.7 ± 9.4	15/19	16WK	Australia	FA;QOL
Santagnello	2020	RET/PB	2-arm RCT	52.1 ± 10.1 59 ± 9.2	11/9	12WK	Brazil	FA
Huo	2024	PNF/MM/PB	3-arm RCT	51.3 ± 11.2 49.5 ± 10.7 50.6 ± 12.4	51/50/61	12WK	China	VAS;GS
Herrero	2006	MM/PB	2-arm RCT	50 ± 5 51 ± 10	8/8	8WK	Spain	QOL
Kilbreath	2012	MM/PC	2-arm RCT	53.5 ± 12.1 51.6 ± 11	81/79	8WK	Australia	QOL
Stone	2012	RET/PB	2-arm RCT	62.3 ± 6.7 62.3 ± 6.7	52/54	24WK	America	QOL;GS
Basha	2022	VR/RET	2-arm RCT	48.83 ± 7 52.07 ± 7.48	30/30	8WK	Egypt	VAS;DASH;QOL;GS
Cho	2016	MLD/PB	2-arm RCT	46.6 ± 6.8 50.7 ± 9.6	21/20	4WK	Korea	VAS;FA;DASH;QOL
Esteban	2024	RET/PB	2-arm RCT	52.6 ± 8.8 52 ± 9.4	32/28	12WK	Spain	DASH
Basoglu	2021	KT/PB	2-arm RCT	53.7 ± 8.6 53.4 ± 8.3	17/19	4WK	Turkey	DASH;GS
Guloglu	2023	PNF/RET/PB	3-arm RCT	46 ± 7.7 48.8 ± 9.8 44.2 ± 7	22/22/22	4WK	Turkey	VAS;DASH
Erden	2022	ET/PB	2-arm RCT	57.1 ± 10.88 56.9 ± 10.2	40/40	4WK	Turkey	VAS
Cornette	2016	MM/PB	2-arm RCT	50.4 ± 8.3 52.85 ± 9.43	20/22	27WK	French	QOL
Casanovas	2024	MM/PB	2-arm RCT	49.2 ± 10.9 54.7 ± 12.1	32/32	12WK	Spain	VAS;FA;QOL;GS
Cakit	2024	AE/PB	2-arm RCT	61.92 ± 12.41 59.23 ± 11.86	15/17	3WK	Turkey	VAS
Ramadan	2024	KT/PB	2-arm RCT	48.95 ± 5.05 51.05 ± 4.27	20/20	12WK	Egypt	QOL
Schmidt	2012	RET/AET	2-arm RCT	58 ± 8.41 55 ± 10.59	15/18	12WK	Germany	FA;QOL
Yuen	2007	RET/AET/PB	3-arm RCT	53.7 ± 11.3 53.1 ± 13.5 55 ± 13.4	7/7/8	12WK	America	FA
Newton	2015	PC/PB	2-arm RCT	61.5 ± 9.2 61.5 ± 9.2	13/11	4WK	Australia	QOL
Ozsoy	2019	KT/PB	2-arm RCT	50.56 ± 6.45 54.52 ± 7.49	16/19	4WK	Turkey	VAS
Mur-Gimeno	2024	ALT/MM	2-arm RCT	58.1 ± 9.5 52.3 ± 9.9	14/14	12WK	Spain	QOL
Toprak	2019	PC/MLD	2-arm RCT	55.36 ± 10.3 59.04 ± 2.83	22/24	5WK	Turkey	QOL
Lin	2023	RET/AET/PB	3-arm RCT	49.38 ± 9.51 47.37 ± 9.99 51.69 ± 10.14	47/48/48	12WK	China	VAS
Kim	2010	RET/PB	2-arm RCT	50.5 ± 10.58 50.9 ± 9.15	20/20	8WK	Korea	QOL
Martina	2015	RET/PB	2-arm RCT	52.2 ± 9.9 53.3 ± 10.2	49/46	12WK	Germany	FA;QOL
Erkan	2020	LLLT/PB	2-arm RCT	51.74 ± 5.29 55.86 ± 3.44	21/21	4WK	Turkey	GS
Tastaban	2019	PC/PB	2-arm RCT	52.48 ± 3.51 54.59 ± 2.34	38/38	4WK	Turkey	VAS;DASH;GS

AE, aquatic exercise; AET, aerobic exercise; ALT, aqua lymphatic therapy; ET, electrotherapy; KT, kinesio taping; LLLT, low level laser therapy; MLD, manual lymphatic drainage; MM, mixed motion; MO, moxibustion; PC, pneumatic circulation; PNF, proprioceptive neuromuscular facilitation; RET, resistance exercise; VR, virtual reality; YG, yoga; UG, ultrasound therapy; SUCAR, Surface Under The Cumulative Ranking Curve; VAS, visual analog scale; GS, Grip strength; FA, Fatigue Severity Scale; QOL, Quality of Life; DASH, Disabilities of Arm, Shoulder and Hand.

**Table 2:** Risk assessment for inclusion studies.

Study	Sequence generation	Allocation concealment	Participant and therapist (Blinding)	Assessor (Blinding)	Incomplete outcome data	Selective reporting	Other bias
Wang 2019	Low risk	Low risk	Unclear	Unclear	Low risk	Low risk	Unclear
Liu 2023	High risk	Low risk	Unclear	Low risk	Low risk	Low risk	Unclear
Lampinen 2021	Low risk	Low risk	Low risk	Unclear	Low risk	Low risk	Unclear
Atef 2020	Low risk	Unclear	Unclear	Unclear	Low risk	Low risk	Unclear
Haines 2010	Low risk	Low risk	Low risk	Unclear	Low risk	Low risk	Unclear
Kilbreat 2020	Low risk	Low risk	Low risk	Low risk	Low risk	Low risk	Unclear
Dayes 2013	Low risk	Unclear	Low risk	Unclear	Low risk	Low risk	Unclear
Meer 2023	Low risk	Unclear	Low risk	Unclear	Low risk	Low risk	Unclear
Moro 2024	Unclear	Unclear	Unclear	Low risk	Low risk	Low risk	Unclear
Letellier 2014	Low risk	Low risk	Unclear	Low risk	Low risk	Low risk	Unclear
Ahmed 2006	Unclear	Low risk	Unclear	Unclear	Low risk	Low risk	Unclear
Feyzioglu 2020	Low risk	Low risk	Unclear	Low risk	Low risk	Low risk	Unclear
Baxter 2018	Low risk	Low risk	Unclear	Low risk	Low risk	Low risk	Unclear
Ridner 2013	Low risk	Low risk	Unclear	Low risk	Low risk	Low risk	Unclear
Ahmed 2011	High risk	Low risk	High risk	Unclear	Low risk	Low risk	Unclear
Kozanoglu 2009	Low risk	Low risk	Unclear	Low risk	Low risk	Low risk	Unclear
Belmonte 2012	Low risk	Low risk	Low risk	Low risk	Low risk	Low risk	Low risk
Song 2020	Low risk	Low risk	Low risk	Low risk	Low risk	Low risk	Unclear
Hemmati 2022	Low risk	Low risk	Low risk	Low risk	Low risk	Low risk	Unclear
Robb 2007	Low risk	Low risk	Low risk	Low risk	Low risk	Low risk	Low risk
Conejo 2018	Low risk	Low risk	Low risk	Low risk	Low risk	Low risk	Low risk
Ergin 2019	Unclear	Unclear	Unclear	Low risk	Low risk	Low risk	Unclear
Tantawy 2019	Low risk	Low risk	Unclear	Low risk	Low risk	Low risk	Unclear
Tsai 2009	Low risk	Low risk	Unclear	Low risk	Low risk	Low risk	Unclear
Melgaard 2016	Low risk	Low risk	Low risk	Low risk	Low risk	Low risk	Unclear
Garcia 2024	Unclear	Unclear	Low risk	Unclear	Low risk	Low risk	Unclear
Gradalski 2015	Low risk	Low risk	Unclear	Low risk	Low risk	Low risk	Unclear
Sen 2021	Low risk	Unclear	Unclear	Low risk	Low risk	Low risk	Unclear
Xiong 2023	Low risk	Unclear	Unclear	Low risk	Low risk	Low risk	Unclear
Uzkeser 2015	High risk	Low risk	High risk	Low risk	Low risk	Low risk	Unclear
Carrera 2024	Low risk	Low risk	Unclear	Low risk	Low risk	Low risk	Unclear
Oliveira 2014	Unclear	Unclear	Unclear	Low risk	Low risk	Low risk	Unclear
Ergin 2017	High risk	Low risk	Unclear	Low risk	Low risk	Low risk	Unclear
Tambour 2018	Low risk	Low risk	Unclear	Low risk	Low risk	Low risk	Unclear
Devoogdt 2011	Low risk	Low risk	Low risk	Low risk	Low risk	Low risk	Unclear
Nele 2018	Low risk	Low risk	Low risk	Low risk	Low risk	Low risk	Unclear
Villanueva 2013	Low risk	Low risk	Unclear	Low risk	Low risk	Low risk	Unclear
Ali 2021	Low risk	Low risk	Unclear	Low risk	Low risk	Low risk	Unclear
McNeely 2004	Unclear	Low risk	Unclear	Low risk	Low risk	Low risk	Unclear
Bahtiyarca 2019	Low risk	Low risk	Unclear	Low risk	Low risk	Low risk	Unclear
Conwright 2021	Low risk	Low risk	Low risk	Low risk	Low risk	Low risk	Unclear
Winters 2022	Low risk	Low risk	Unclear	Low risk	Low risk	Low risk	Unclear
Milne 2008	Low risk	Unclear	Unclear	Low risk	Low risk	Low risk	Unclear
Courneya 2007	Low risk	Unclear	Unclear	Low risk	Low risk	Low risk	Unclear
Sweeney 2019	Low risk	Low risk	Unclear	Low risk	Low risk	Low risk	Unclear
Chaoul 2018	Low risk	Low risk	Unclear	Low risk	Low risk	Low risk	Unclear
Yagli 2015	Unclear	Low risk	Unclear	Low risk	Low risk	Low risk	Unclear
Porter 2019	Low risk	Low risk	Low risk	Low risk	Low risk	Low risk	Unclear
Eyigor 2018	Low risk	Low risk	Unclear	Low risk	Low risk	Low risk	Unclear
Pasyar 2019	High risk	Low risk	Unclear	Unclear	Low risk	Low risk	Unclear
Loudon 2016	Low risk	Low risk	Low risk	Low risk	Low risk	Low risk	Unclear
Vadiraja 2017	Low risk	Low risk	Low risk	Low risk	Low risk	Low risk	Unclear
Bower 2012	Low risk	Low risk	Low risk	Low risk	Low risk	Low risk	Unclear
Banasik 2011	Unclear	Unclear	Unclear	Unclear	Low risk	Low risk	Unclear
Jong 2018	Low risk	Low risk	Low risk	Low risk	Low risk	Low risk	Unclear
Wong 2024	Low risk	Low risk	Low risk	Low risk	Low risk	Low risk	Unclear
Moadel 2007	Unclear	Unclear	Unclear	Unclear	Low risk	Low risk	Unclear
Lotzke 2016	Low risk	Low risk	Unclear	Low risk	Low risk	Low risk	Unclear
Vadiraja 2009	Low risk	Low risk	Unclear	Low risk	Low risk	Low risk	Unclear
Chandwani 2014	Low risk	Low risk	Unclear	Low risk	Low risk	Low risk	Unclear
Hosakote 2009	Low risk	Low risk	Unclear	Low risk	Low risk	Low risk	Unclear
Siedentofy 2013	High risk	Low risk	High risk	Low risk	Low risk	Low risk	Unclear
Taso 2014	Low risk	Low risk	Unclear	Low risk	Low risk	Low risk	Unclear
Cramer 2015	Low risk	Low risk	Unclear	Low risk	Low risk	Low risk	Unclear
Vardar 2015	Unclear	Low risk	Unclear	Low risk	Low risk	Low risk	Unclear
Stan 2016	Low risk	Low risk	Unclear	Low risk	Low risk	Low risk	Unclear
Littman 2012	Low risk	Low risk	Unclear	Low risk	Low risk	Low risk	Unclear
Annette 2014	Low risk	Low risk	Unclear	Low risk	Low risk	Low risk	Unclear
Dahhak 2022	Low risk	Low risk	Low risk	Low risk	Low risk	Low risk	Unclear
Naczk 2022	Low risk	Low risk	Low risk	Low risk	Low risk	Low risk	Unclear
Michels 2023	Low risk	Low risk	Unclear	Low risk	Low risk	Low risk	Unclear
Husebo 2014	Low risk	Low risk	High risk	Low risk	Low risk	Low risk	Unclear
Buchan 2016	Low risk	Low risk	Unclear	Low risk	Low risk	Low risk	Unclear
Singh 2016	Low risk	Unclear	Unclear	Low risk	Low risk	Low risk	Unclear
Paulo 2019	Low risk	Unclear	Unclear	Low risk	Low risk	Low risk	Unclear
Taradaj 2016	Low risk	Unclear	Unclear	Low risk	Low risk	Low risk	Unclear
Liu 2022	Low risk	Low risk	Low risk	Low risk	Low risk	Low risk	Unclear
Lee 2022	Unclear	Unclear	Unclear	Unclear	Low risk	Low risk	Unclear
Cormie 2013	Low risk	Low risk	Unclear	Low risk	Low risk	Low risk	Unclear
Soidan 2020	Low risk	Low risk	Low risk	Low risk	Low risk	Low risk	Unclear
Bloomquist 2021	Low risk	Low risk	Unclear	Low risk	Low risk	Low risk	Unclear
Omar 2020	Low risk	Low risk	Low risk	Low risk	Low risk	Low risk	Unclear
Steindorf 2014	Low risk	Low risk	Low risk	Low risk	Low risk	Low risk	Unclear
Park 2023	Unclear	Unclear	Unclear	Unclear	Low risk	Low risk	Unclear
Hagstrom 2016	Low risk	Low risk	Low risk	Low risk	Low risk	Low risk	Unclear
Santagnello 2020	Low risk	Low risk	Unclear	Unclear	Low risk	Low risk	Unclear
Huo 2024	Low risk	Low risk	Low risk	Low risk	Low risk	Low risk	Unclear
Herrero 2006	Low risk	Low risk	Unclear	Low risk	Low risk	Low risk	Unclear
Kilbreath 2012	Low risk	Low risk	Unclear	Low risk	Low risk	Low risk	Unclear
Stone 2012	Low risk	Low risk	Unclear	Low risk	Low risk	Low risk	Unclear
Basha 2022	Low risk	Low risk	Unclear	Low risk	Low risk	Low risk	Unclear
Cho 2016	Unclear	Unclear	Unclear	Unclear	Low risk	Low risk	Unclear
Esteban 2024	Low risk	Low risk	Unclear	Low risk	Low risk	Low risk	Unclear
Basoglu 2021	Low risk	Unclear	Unclear	Unclear	Low risk	Low risk	Unclear
Guloglu 2023	Unclear	Unclear	High risk	Unclear	Low risk	Low risk	Unclear
Erden 2022	Low risk	Low risk	Unclear	Low risk	Low risk	Low risk	Unclear
Cornette 2016	Unclear	Unclear	Unclear	Unclear	Low risk	Low risk	Unclear
Casanovas 2024	Low risk	Low risk	Unclear	Low risk	Low risk	Low risk	Unclear
Cakit 2024	Low risk	Unclear	Unclear	Low risk	Low risk	Low risk	Unclear
Ramadan 2024	Low risk	Low risk	Unclear	Low risk	Low risk	Low risk	Unclear
Schmidt 2012	Low risk	Unclear	Unclear	Low risk	Low risk	Low risk	Unclear
Yuen 2007	Low risk	Unclear	Unclear	Low risk	Low risk	Low risk	Unclear
Newton 2015	Low risk	Unclear	High risk	Low risk	Low risk	Low risk	Unclear
Ozsoy 2019	Low risk	Low risk	Unclear	Low risk	Low risk	Low risk	Unclear
Mur-Gimeno 2024	Low risk	Low risk	Unclear	Low risk	Low risk	Low risk	Unclear
Toprak 2019	Low risk	Unclear	Unclear	Unclear	Low risk	Low risk	Unclear
Lin 2023	Low risk	Low risk	Unclear	Low risk	Low risk	Low risk	Unclear
Kim 2010	High risk	Unclear	High risk	Unclear	Low risk	Low risk	Unclear
Martina 2015	Unclear	Unclear	High risk	Unclear	Low risk	Low risk	Unclear
Erkan 2020	Low risk	Unclear	Unclear	Unclear	Low risk	Low risk	Unclear
Tastaban 2019	Low risk	Low risk	Unclear	Low risk	Low risk	Low risk	Unclear

### Results of network meta-analysis

#### Network diagram

**Figure 2:**
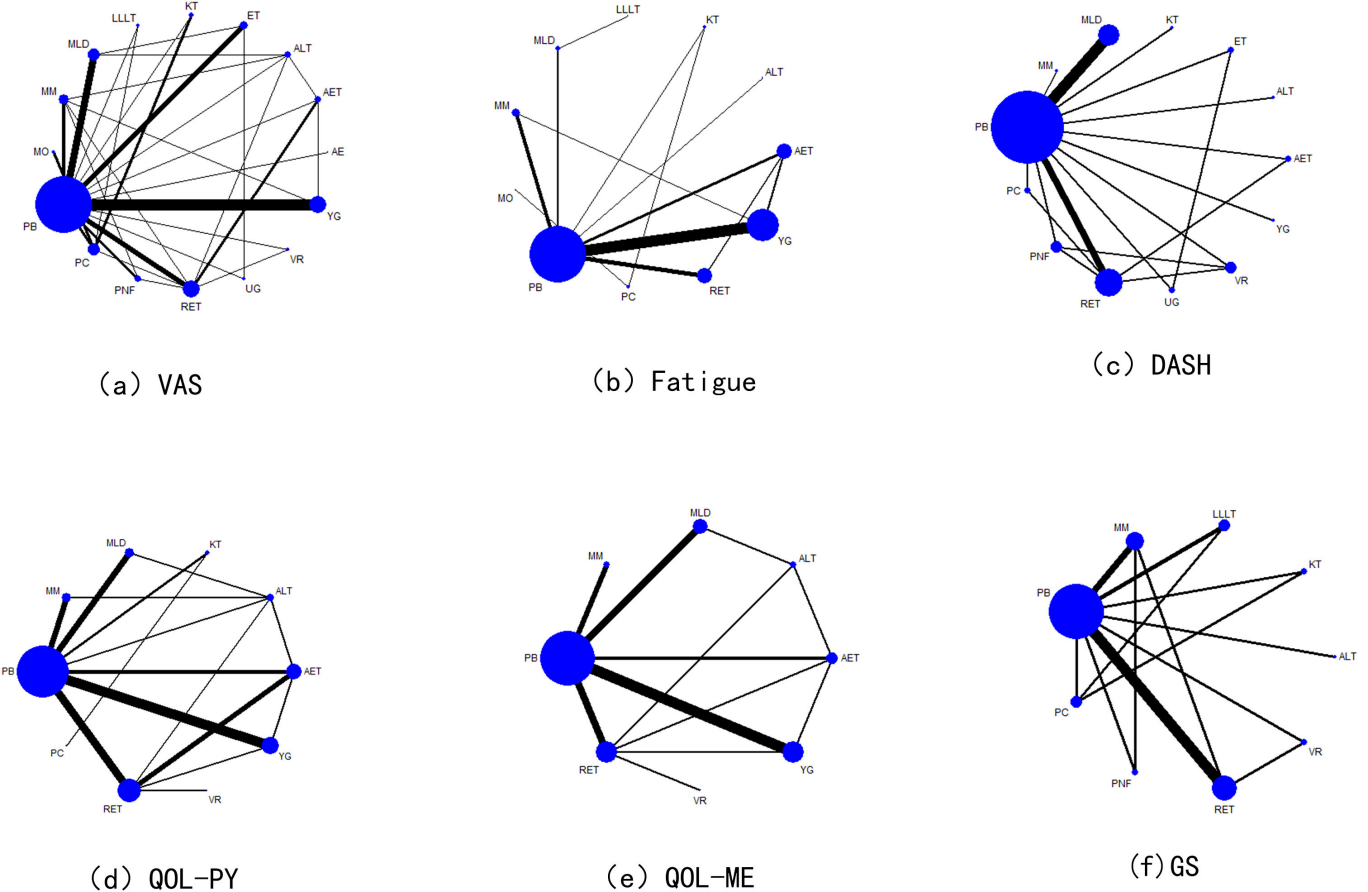
Network diagram.

A total of 16 interventions were included in the literature review, of which 47 studies reported VAS, 38 studies reported Fatigue Assessment, 22 studies reported DASH(Disabilities of Arm, Shoulder and Hand) Functional Disability Index, and 36 studies reported QOL (Physical Component) scores. QOL (Mental Component) scores were reported in 26 studies, and GS scores were reported in 18 studies. The results are illustrated in **Figure 2**.

### Inconsistency testing and reliability testing

For all outcome measures that constitute network evidence, no significant inconsistencies were detected, thereby substantiating the hypothesis that network analysis possesses satisfactory internal consistency. A web meta-analysis of confidence in CINeMA was employed to assess confidence, and the overall quality of evidence was found to be substandard (see **Appendix** for details).

### Analysis of the results of each index

#### Pain assessment

**Figure 3:**
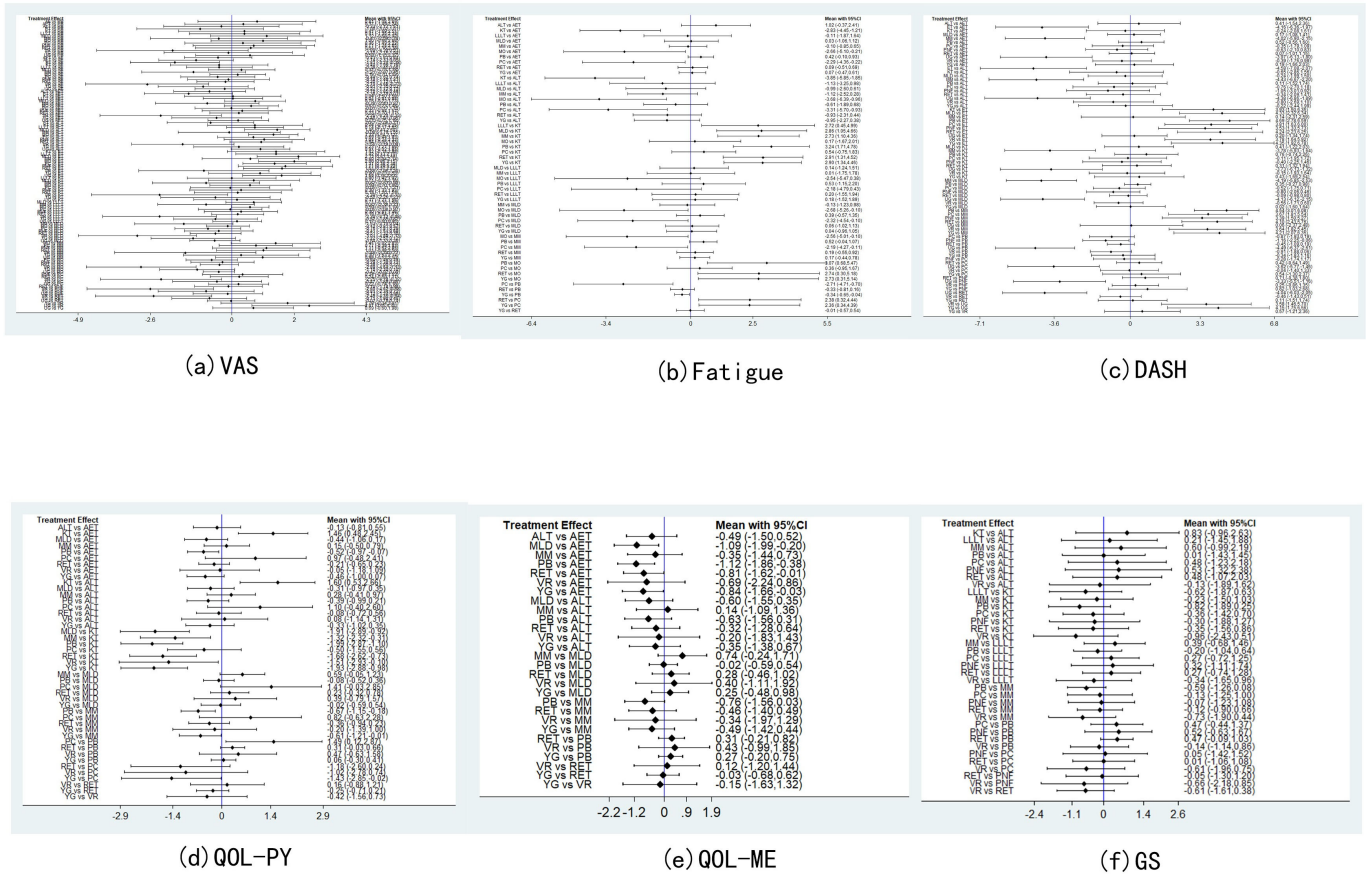
Forest plot.

**Figure 4:**
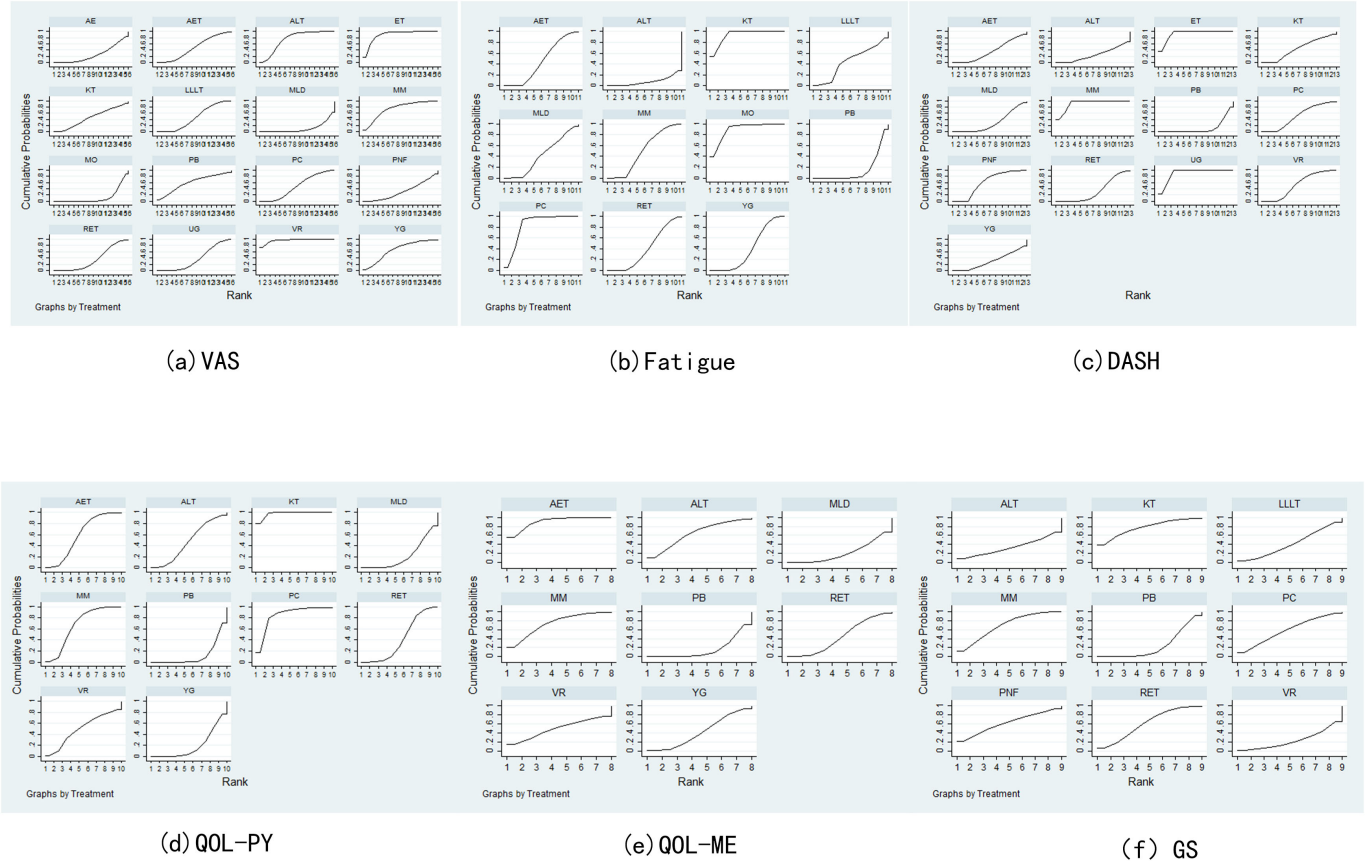
Sucar rank chart.

Pain assessment was reported in 47 studies: Virtual Reality was associated with significantly lower pain scores compared to Aerobic Exercise (SMD = –2.26, 95% CI: –4.20 to –0.33). Electrotherapy also showed superior pain reduction relative to Aquatic Exercise (SMD = –1.81, 95% CI: –3.24 to –0.38). In contrast, Manual Lymphatic Drainage resulted in significantly higher pain scores compared to Aqua Lymphatic Therapy (SMD = 1.45, 95% CI: 0.41 to 2.50), and Low-Level Laser Therapy was associated with higher pain scores relative to Electrotherapy (SMD = 1.42, 95% CI: 0.13 to 2.70). The SUCRA value of Virtual Reality (96.5%) was relatively high, followed by Electrotherapy (89.4%) and Aqua Lymphatic Therapy (75.3%).As demonstrated in **Figures 3a**, **4a**.

#### Fatigue assessment

Thirty-eight studies reported fatigue assessment data: Placebo was associated with significantly higher fatigue scores compared to Kinesio Taping (SMD = 3.24, 95% CI: 1.71 to 4.78) and Moxibustion (SMD = 3.07, 95% CI: 0.68 to 5.47). In contrast, Pneumatic Circulation demonstrated significantly lower fatigue scores than Placebo (SMD = –2.71, 95% CI: –4.71 to –0.70), as did Yoga (SMD = –0.34, 95% CI: –0.65 to –0.04). The SUCRA analysis revealed that Kinesio Taping (93.5%) has a more significant effect, followed by Moxibustion (89.9%) and Pneumatic Circulation (83.6%). As illustrated in **Figures 3b**, **4b**.

### DASH functional disability

Twenty-two studies reported DASH functional disability index: Placebo was associated with significantly higher disability scores compared to Electrotherapy (SMD = 4.69, 95% CI: 2.78 to 6.59) and Mixed Motion (SMD = 4.54, 95% CI: 3.01 to 6.08). Conversely, Proprioceptive Neuromuscular Facilitation resulted in significantly lower scores than Placebo (SMD = –1.16, 95% CI: –2.25 to –0.06), as did Ultrasound therapy (SMD = –4.49, 95% CI: –6.37 to –2.61). The SUCRA analysis revealed that Electrotherapy (93%) has a more significant effect, followed by Mixed Motion (91.2%) and Ultrasound therapy (90.7%). As demonstrated in **Figures 3c**, **4c**.

### QOL (Physical component)

Thirty-six studies reported QOL (Physical component): Placebo was associated with significantly lower QOL scores compared to Aerobic Exercise (SMD = –0.52, 95% CI: –0.97 to –0.07), Kinesio Taping (SMD = –1.99, 95% CI: –2.87 to –1.10), and Mixed Motion (SMD = –0.67, 95% CI: –1.15 to –0.18). Kinesio Taping (97.8%) had a more favorable effect on SUCRA, followed by Pneumatic Circulation (84.7%) and Mixed Motion (67.3%). As demonstrated in **Figures 3d**, **4d**.

### QOL (Mental component)

Twenty-six studies reported QOL (Mental component): Placebo was associated with significantly lower QOL scores compared to Aerobic Exercise (SMD = –1.12, 95% CI: –1.86 to –0.38). Similarly, Manual Lymphatic Drainage demonstrated significantly lower scores relative to Aerobic Exercise (SMD = –1.09, 95% CI: –1.99 to –0.20), as did Resistance Exercise (SMD = –0.81, 95% CI: –1.62 to –0.01). The SUCRA value of Aerobic Exercise (90.3%) was relatively strong, followed by Mixed Motion (73%) and Aqua Lymphatic Therapy (63.7%). As demonstrated in **Figures 3e**, **4e**.

### Grip strength

The results of the network meta-analysis for grip strength (GS), based on 18 studies, indicated that no significant differences were observed between Placebo and Aqua Lymphatic Therapy (SMD = –0.01, 95% CI: –1.43 to 1.45), Kinesio Taping (SMD = –0.82, 95% CI: –1.89 to 0.25), or Mixed Motion (SMD = –0.59, 95% CI: –1.26 to 0.08). The SUCRA value of Kinesio Taping (77.2%) was relatively high, followed by Mixed Motion (68.4%) and Resistance Exercise (61.5%). As demonstrated in **Figures 3f**, **4f**.

### Publication bias

**Figure 5:**
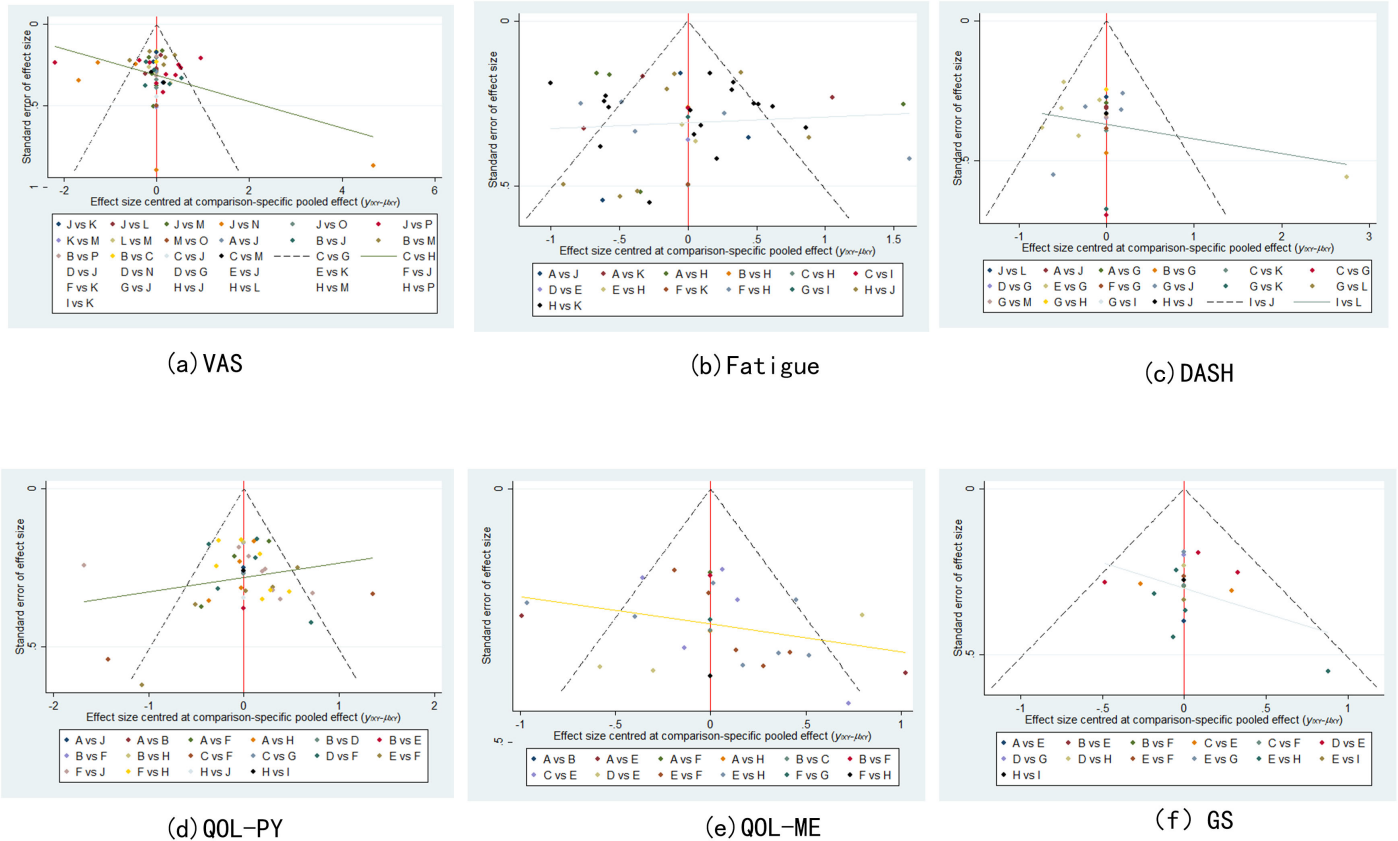
Funnel plot.

Correction-comparison funnel plots of VAS, fatigue, DASH, QOL (physical component), QOL (mental component), and GS were plotted to assess publication bias. It can be seen that all points basically fall within the funnel, and the distribution of scatter points on both sides of X = 0 is roughly symmetrical, suggesting that the possibility of publication bias or small sample effect is small (see **Figure 5**).

## Discussions

In this systematic review and network meta-analysis of randomized controlled trials, the effect of various physical therapies on breast cancer survivors was found to be positive in comparison to placebo (home schooling or primary care). However, the evidence results were moderate, either by themselves or in combination with other medications or surgery. We believe that based on the SUCRA assessment, VR is a relatively effective physical therapy method in terms of pain improvement. The analgesic mechanism of Virtual Reality (VR) is primarily attributed to its capacity to engage multiple attentional and cognitive resources, thereby diverting processing capacity away from nociceptive signals in a manner consistent with the limited capacity model of attention (67). In terms of improving fatigue scores, kinesiology tape may be more effective. It is believed to be able to promote local microcirculation and the drainage of lymph fluid, thereby helping to eliminate metabolic waste and improve the oxygen supply to tissues. At the same time, the neuro-regulatory effect produced by continuous skin stimulation may help restore the abnormal muscle tension to normal levels and reduce pain through the gating theory, thereby alleviating fatigue conditions (137, 138). However, for DASH functional disability, electrotherapy may be a more effective form of physical therapy. This might be achieved through its various neuroregulatory and physiological effects, by activating large-diameter afferent fibers to “gate control” the transmission of nociceptive signals in the spinal cord, and possibly stimulating the release of endogenous opioids, thereby regulating pain perception. Furthermore, electrotherapy helps prevent muscle atrophy and enhance local blood circulation, thereby addressing potential damage to muscle function and promoting tissue recovery. This combined effect of pain relief and recovery alleviates pain and facilitates the functional use of the upper limbs (85). Due to the predominance of female subjects in the study, a gender-based subgroup analysis was not feasible. However, a preliminary investigation into age stratification revealed a correlation between younger age and greater benefit. The intensity of physical therapy cannot be fully assessed in survivors of different stages of breast cancer. However, mixed exercise has been shown to have some advantages in terms of selection as adjuvant therapy for breast cancer. An appropriate increase in exercise intensity may be more conducive to the improvement of patient function. This is consistent with the views of Zhou et al. (139) that mixed exercise and resistance exercise can effectively improve the fatigue experienced by breast cancer survivors. Furthermore, it has been demonstrated that exercise intervention with a frequency of ≥3 times per week, lasting > 60 minutes each time and > 180 minutes per week, has a more pronounced effect. Increasing the level of physical activity has been shown to reduce the risk of various cancers, and the appropriate intensity of exercise can effectively reduce the overall mortality and adverse reactions of various cancers, including breast cancer (139–142). It is imperative to raise awareness of the benefits of exercise and to conduct disease screening and assessment according to factors such as age and gender, which can effectively reduce the medical burden (143). In a similar vein, it is postulated that the incorporation of physical therapy modalities such as pneumatic circulation therapy, Aqua lymphatic therapy, aerobic exercise and mixed exercise into the therapeutic regimen of breast cancer survivors would prove to be of considerable benefit in enhancing their quality of life.

Our review did not find an exact causal mechanism, but the statistical findings are valid. A single mechanism of action may not fully explain all of our findings. We therefore considered a number of hypotheses, including a combination of mindfulness or psychological cues (144), competing mechanisms (145), interstitial or lymphatic regulation (146), edema blocking mechanisms (147), neuromuscular regulation (18), functional or pain-related (148), and photobiological regulation (149), to produce a positive and favorable outcome. The meta-findings found that these factors were associated with improved quality of life or lymphedema in breast cancer survivors, but could not fully explain why a single mechanism covered all factors. Yoga exercises, for example, can directly promote the role of mindfulness, while also improving mental health (150); Various kinds of sports, including aerobic exercise, resistance exercise and mixed exercise, they have a certain competitive relationship, but also affect each other, because no sport can exist completely independently (151–153); The effects of pressure therapy, bandages and kinesio taping on edema blockage in breast cancer survivors were profound (123, 154, 155). Proprioceptive neuromuscular facilitation technology and virtual reality technology are important manifestations of neuromuscular regulation (156, 167). Electrotherapy, moxibustion, and ultrasound therapy provide more positive effects on pain and function (29, 157, 158). Manual lymphatic drainage may satisfy a variety of mechanisms, but it cannot cover all aspects (66, 159). We believe that understanding the mechanisms of action of these treatments can lead to better understanding and development of adjuvant treatment plans.

Our review included more studies than previous reviews of breast cancer survivors with various physical therapies (148, 149, 160–164). Therefore, we can draw a more comprehensive and accurate conclusion. We included 111 studies for statistical analysis, and the confidence interval is narrower than that of most existing meta-analyses, and the accuracy of the estimation is higher (165). At the same time, we found a significant phenomenon that for the treatment cycle, the shorter the time, the better the effect, which was similar to the study results of Wahid et al. (164). However, this is not absolute, because most statistics do not have an absolute linear time sequence, we cannot give a precise judgment on the duration of the treatment effect, and it is certain that the long-term (beyond 12 months) effect after treatment is gradually reduced. In our review, some niche treatments, such as the use of intramuscular patches, had good results, possibly because mesh meta-analyses used smaller study data with higher efficacy than ordinary meta-analyses. In addition, a proportion of the studies we included combined different interventions, making it more difficult to interpret the estimates of meta-analyses.

This study shows that virtual reality technology can improve the pain of breast cancer survivors most obviously. This non-invasive, non-pharmaceutical choice may be based on the fact that virtual reality can distract patients’ attention and reduce their pain experience to a certain extent through meditation and mindfulness technology (166–168). The use of virtual reality technology in clinical practice is not uncommon, whether in assessment or treatment, immersive gaming experience and emotional rendering, which is also effective for mental illness in breast cancer survivors (169–171). Aerobic exercise is very effective in improving the psychological aspect of quality of life, and the importance of exercise for cancer patients has been generally emphasized. Regular exercise can improve physical function, enhance the immune function of cancer patients, psychologically provide better feelings and reduce stress, depression and anxiety (172–175). In addition, we found good acceptance of electrotherapy, manual lymphatic drainage, and pneumatic circulation therapy, due to a lower percentage of dropouts or omissions found in most of the included studies, although measurements of dropouts are not fully representative of patient acceptance. Whether a patient completes the study depends largely on the interest and effectiveness of the adjuvant treatment program. Of course, these passive physical therapies seem to be more satisfying to patients. However, we are confused that the opt-out rate in the control group is still not high, and there are many included literatures that do not mention these useful data, so more high-quality studies are needed to confirm these results.

The present study is subject to several limitations. Firstly, the literature included is all in English, which may result in geographical and ethnic bias, although the comparison of adjusted funnel plots suggests that this probability is not high. Secondly, the large number of included studies may have resulted in heterogeneity due to differences in research objects, intervention measures, outcome indicators, etc. Despite the implementation of stricter inclusion criteria and quality assessment, these heterogeneities could not be eliminated. For instance, when assessing the quality of life, not all relevant scales were included. We mainly incorporated assessment tools such as SF-36 (Short Form 36 Health Survey) and EORTC QLQ (European Organization for Research and Treatment of Cancer Quality of Life Questionnaire), which led to the exclusion of some quality assessment tools like ULL-27 (Upper Limb Lymphedema-27). Vatansever et al. demonstrated that the ULL-27 questionnaire is a reliable and effective scale for assessing the quality of life of patients with upper limb lymphedema (176). In addition, the treatment methods and treatment cycles of the included studies exhibited significant heterogeneity, and the disease progression of patients was not completely consistent. The standards of resistance exercise, aerobic exercise and mixed exercise in exercise therapy were not fully unified, which may also be the cause of large heterogeneity. Low confidence levels are usually due to in-study bias, imprecise treatment effects, or lack of randomization and assignment of hidden information (21). Given that a significant portion of the included randomized controlled trials were assessed as having “low” or “very low” confidence levels based on the CINeMA evaluation, these findings must be interpreted with caution. The inherent limitations of this primary evidence significantly weaken the strength and generalizability of our conclusions, and emphasize that they should not be regarded as direct clinical application guidelines without further validation. Consequently, there is a necessity for further high-quality, multi-center, large-sample studies to be conducted in the future in order to strengthen the data.

In light of the significance of clinical decision-making, there is a need to elucidate the benefits and limitations of employing diverse physical therapy interventions in the management of breast cancer survivors. This information should be made readily available to physicians, rehabilitation therapists, and caregivers. The findings of this study should contribute to the development of future guidelines or the revision of existing information, with the objective of ensuring that patients receive optimal physical therapy and care. The results of our network meta-analysis show that all physical therapy measures seem to be effective compared with the placebo group. This finding has considerable value in clinical practice.

## Conclusion

All physical therapy measures demonstrated efficacy in breast cancer survivors when compared with placebo. Virtual reality technology exhibited the most significant effect on pain improvement, while electrotherapy demonstrated the most substantial effect on functional disability recovery. Intramuscular tape exhibited the most marked effect on fatigue, physical quality of life and grip strength, and aerobic exercise exhibited the most substantial effect on psychological quality of life. However, these findings require further validation through large-scale, multicenter, randomized controlled trials (RCTs).

## Data Availability

The original contributions presented in the study are included in the article/**Supplementary Material**. Further inquiries can be directed to the corresponding author.
