# Supplementary material for: Comparative efficacy of various physical therapies on pain, fatigue, quality of life and functional impairment in breast cancer survivors: a network meta-analysis of randomized controlled trials
Source: Front Oncol. 2025 Dec 12;15:1699682. doi: 10.3389/fonc.2025.1699682 (PMC12740869; doi:10.3389/fonc.2025.1699682)
Supplement: Supplementary file 1 [file DataSheet1.doc]

Efficacy of different physical therapy modalities in breast cancer survivors: a network meta-analysis based on randomized clinical trials

Search Strategy

**Pubmed：1168**

**(random) AND ((((((((((((((((((((((((((((((((kinesiology tape) OR (tape)) OR (taping)) OR (elastic taping)) OR (kinesio Taping)) OR (kinesiotape)) OR (Manual Lymph Drainage)) OR (Lymphatic Drainage Massage)) OR (low-level laser therapy)) OR (LLLT)) OR (cold laser)) OR (laser)) OR (Intermittent pneumatic compression pump)) OR (barotherapy)) OR (air pressure)) OR (progressive resistance training)) OR (resistance training)) OR (resistance exercise)) OR (strength training)) OR (shockwave)) OR (ESWT)) OR (Shock Wave Therapy)) OR (Electrotherapy)) OR (Aqua lymphatic therapy)) OR (Aquatic exercise)) OR (Aqua Therapy Exercises)) OR (moxibustion therapy)) OR (moxa-moxibustion)) OR (moxibustion)) OR (yoga)) OR ((((("Manual Lymphatic Drainage"[Mesh]) OR "Low-Level Light Therapy"[Mesh]) OR "Extracorporeal Shockwave Therapy"[Mesh]) OR "Electric Stimulation Therapy"[Mesh]) OR "Hydrotherapy"[Mesh])) AND (((breast cancer) OR (Breast cancer-related lymphedema)) OR (("Breast Cancer Lymphedema"[Mesh]) OR "Breast Neoplasms"[Mesh])))**

**Cochrane library：2063**

**#1 MeSH descriptor: [Breast Neoplasms] explode all trees 967**

**#2 (Lymphedema):ti,ab,kw OR (Breast Cancer Lymphedema):ti,ab,kw OR (breast cancer):ti,ab,kw OR (Breast cancer-related lymphedema):ti,ab,kw 48624**

**#3 #1 OR #2 48768**

**#4 MeSH descriptor: [Manual Lymphatic Drainage] explode all trees 41**

**#5 MeSH descriptor: [Low-Level Light Therapy] explode all trees 1732**

**#6 MeSH descriptor: [Extracorporeal Shockwave Therapy] explode all trees 309**

**#7 MeSH descriptor: [Electric Stimulation Therapy] explode all trees 11565**

**#8 MeSH descriptor: [Hydrotherapy] explode all trees 1979**

**#9 (kinesiology tape):ti,ab,kw OR (tape):ti,ab,kw OR (taping):ti,ab,kw OR (elastic taping):ti,ab,kw OR ("Kinesio taping"):ti,ab,kw 7649**

**#10 (kinesiotape):ti,ab,kw OR (Manual Lymph Drainage):ti,ab,kw OR (Lymphatic Drainage Massage):ti,ab,kw OR (low-level laser therapy):ti,ab,kw OR (LLLT):ti,ab,kw 4768**

**#11 (cold laser):ti,ab,kw OR (laser):ti,ab,kw OR (Intermittent pneumatic compression pump):ti,ab,kw OR (barotherapy):ti,ab,kw OR (air pressure):ti,ab,kw 31468**

**#12 (progressive resistance training):ti,ab,kw OR (resistance training):ti,ab,kw OR (resistance exercise):ti,ab,kw OR (strength training):ti,ab,kw OR (shockwave):ti,ab,kw 38919**

**#13 (ESWT):ti,ab,kw OR ("shock-wave therapy"):ti,ab,kw OR (Electrotherapy):ti,ab,kw OR (Aqua lymphatic therapy):ti,ab,kw OR (Aquatic exercise):ti,ab,kw 3941**

**#14 (Aqua Therapy Exercises):ti,ab,kw OR (moxibustion therapy):ti,ab,kw OR (moxa-moxibustion):ti,ab,kw OR (moxibustion):ti,ab,kw OR (yoga):ti,ab,kw 9058**

**#15 #4 OR #5 OR #6 OR #7 OR #8 OR #9 OR #10 OR #11 OR #12 OR #13 OR #14 101133**

**#16 #3 AND #15 2063**

**Web of science 314**

**#1 Breast Neoplasms** (Topic) or **Breast Cancer Lymphedema** (Topic) or **breast cancer** (Topic) or **Breast cancer-related lymphedema** (Topic) and **Preprint Citation Index** (Exclude – Database)

#2 **kinesiology tape** (Topic) or **tape** (Topic) or **elastic taping** (Topic) or **kinesio taping** (Topic) or **kinesiotape** (Topic) or **Manual Lymphatic Drainage** (Topic) or **Manual Lymph Drainage** (Topic) or **Lymphatic Drainage Massage** (Topic) or **Low-Level Light Therapy** (Topic) or **low-level laser therapy** (Topic) or **LLLT** (Topic) or **cold laser** (Topic) or **laser** (Topic) or **Intermittent pneumatic compression pump** (Topic) or **barotherapy** (Topic) or **air pressure** (Topic) or **progressive resistance training** (Topic) or **resistance training** (Topic) or **resistance exercise** (Topic) or **strength training** (Topic) or **Extracorporeal Shockwave Therapy** (Topic) or **shockwave** (Topic) or **ESWT** (Topic) or **Shock Wave Therapy** (Topic) or **Electric Stimulation Therapy** (Topic) or **Electrotherapy** (Topic) or **hydrotherapy** (Topic) or **Aqua lymphatic therapy** (Topic) or **Aquatic exercise** (Topic) or **Aqua Therapy Exercises** (Topic) or **moxibustion therapy** (Topic) or **moxa-moxibustion** (Topic) or **moxibustion** (Topic) or **yoga** (Topic) and **Preprint Citation Index** (Exclude – Database)

#3 (#1 AND #2) AND TS=(random) 314

**Network plots**

1. **Pain score network map**

**Number of studies＝47**

**Number of interventions (including placebo)＝16**

**Number of participants＝2884**


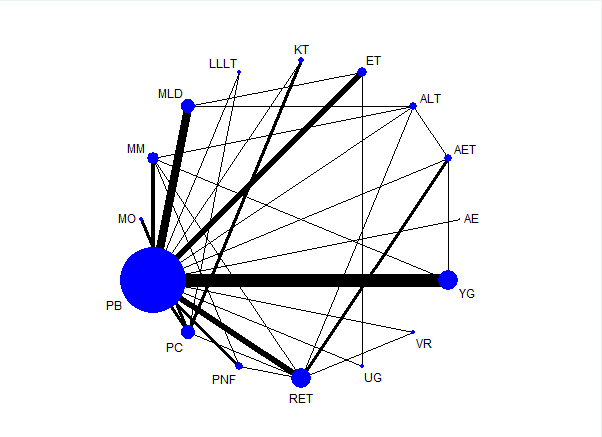


**2.Fatigue assessment network map**

**Number of studies＝39**

**Number of interventions (including placebo)＝11**

**Number of participants＝2537**


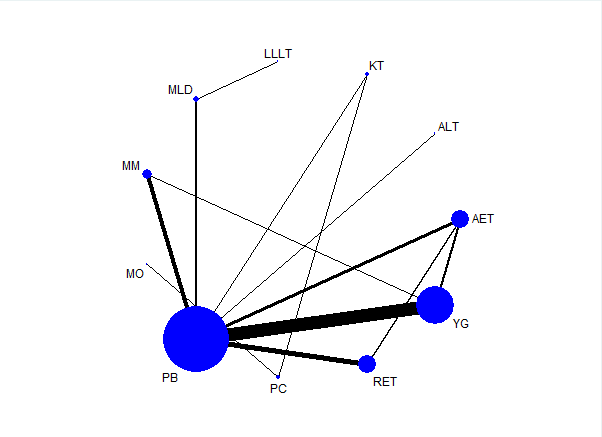


**3.Disabilities of Arm, Shoulder and Hand Assessment Network map of assessment**

**Number of studies＝22**

**Number of interventions (including placebo)＝13**

**Number of participants＝1024**


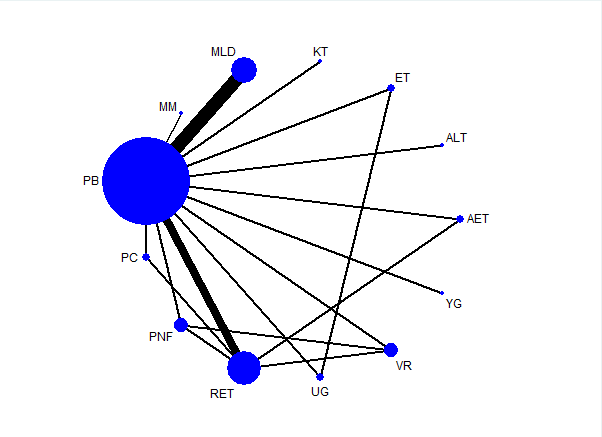


**4.Quality of life (physical components) network map**

**Number of studies＝36**

**Number of interventions (including placebo)＝10**

**Number of participants＝2432**


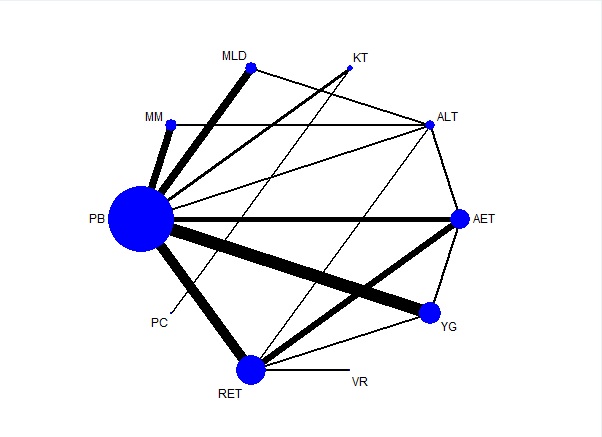


**5.Quality of life (mental components) network map**

**Number of studies＝26**

**Number of interventions (including placebo)＝8**

**Number of participants＝1990**


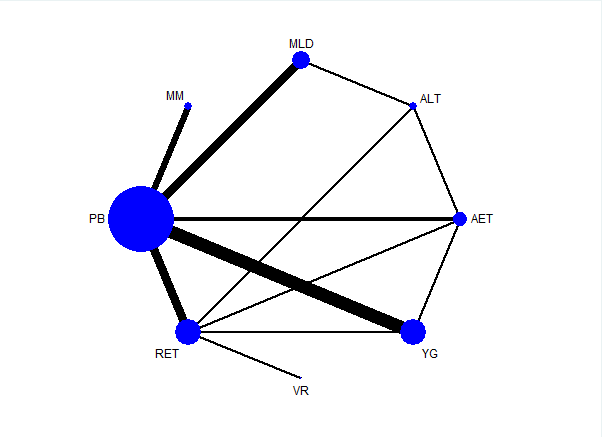


**6.**Grip strength **network map**

**Number of studies＝18**

**Number of interventions (including placebo)＝9**

**Number of participants＝963**


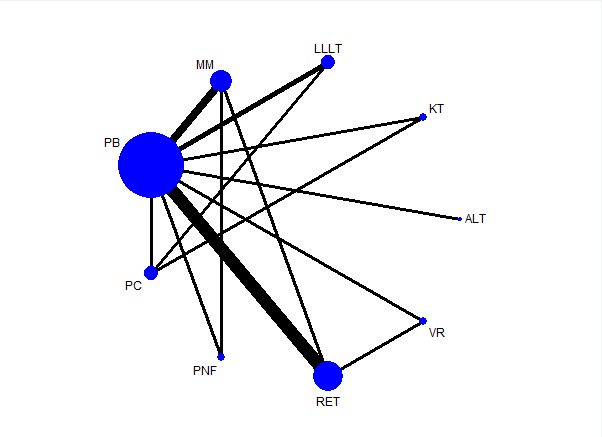


**Forest plots**

**Network meta-analysis: random effects**

1. **Pain assessment forest map**


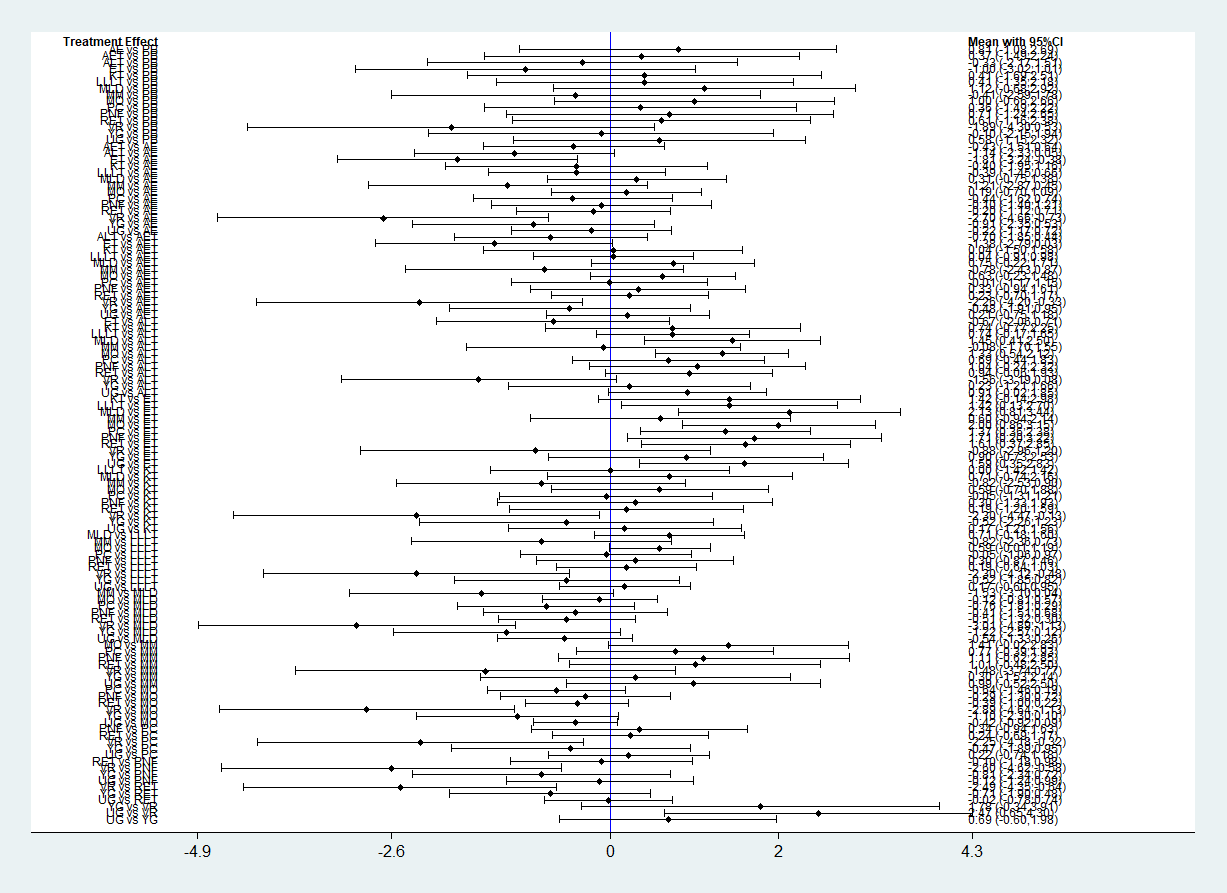


1. **Fatigue assessment forest map**


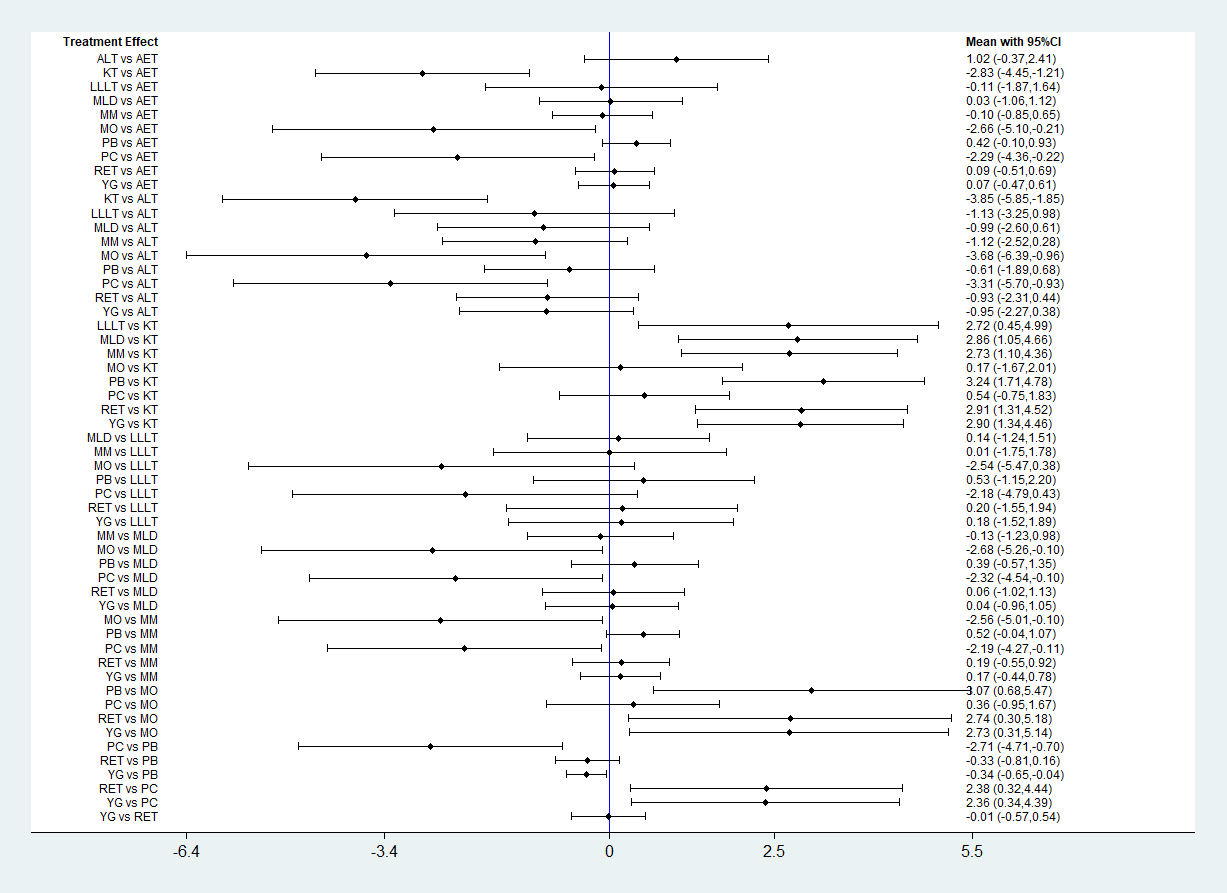


1. **Disabilities of Arm, Shoulder and Hand forest map**


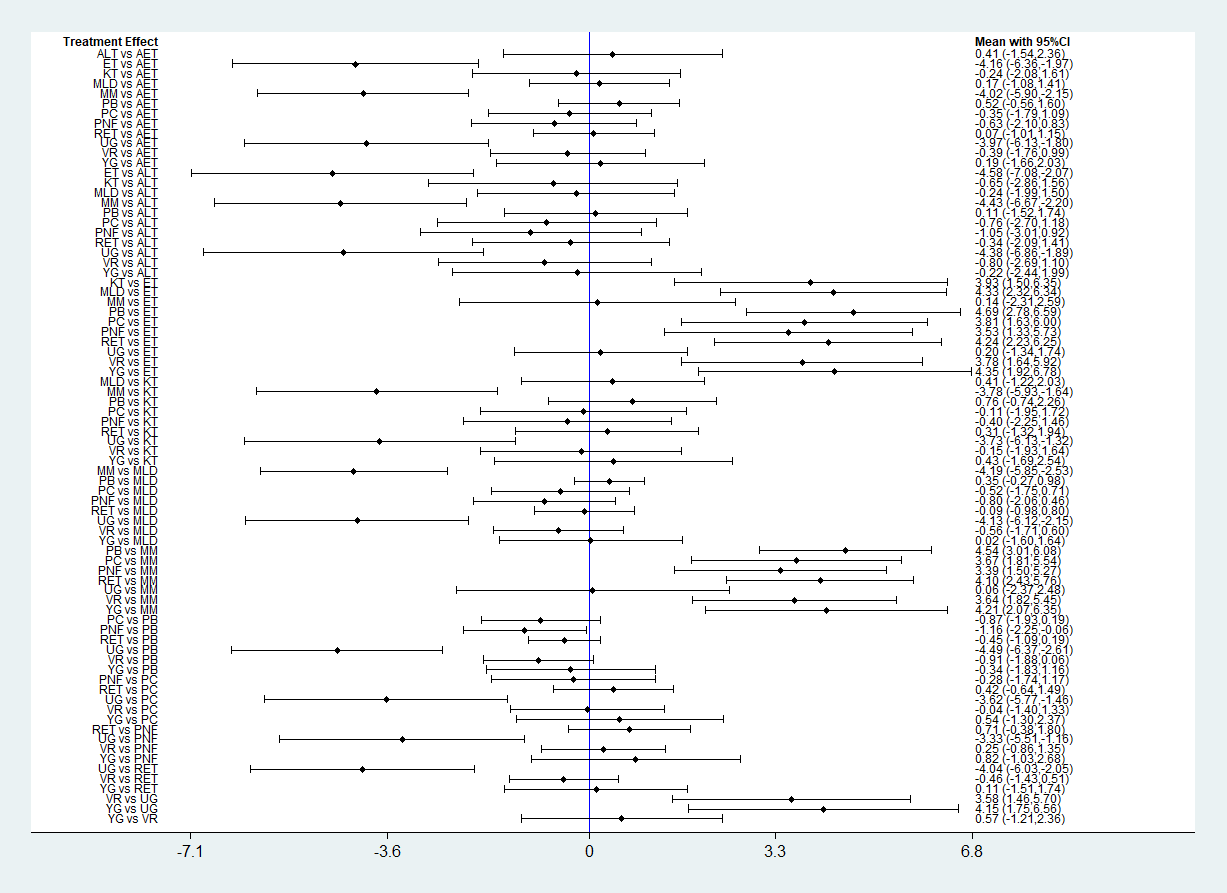


1. **Quality of life (physical components) forest map**


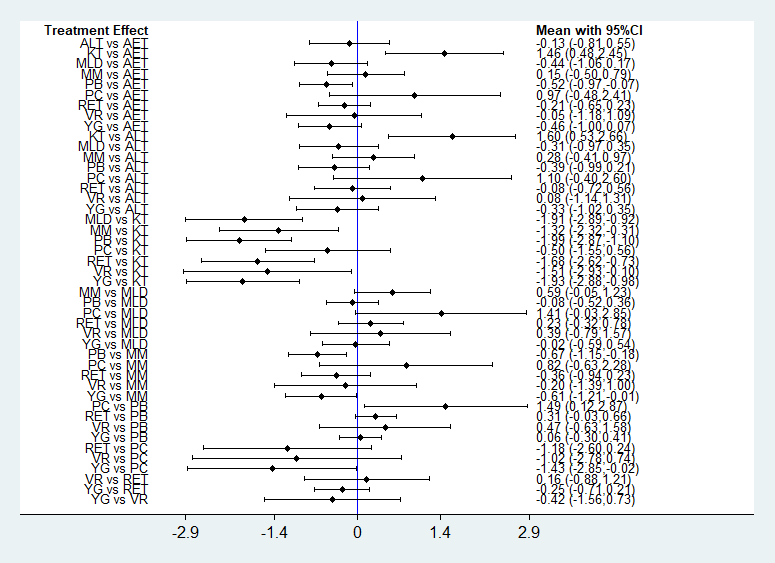


1. **Quality of life (mental components) forest map**


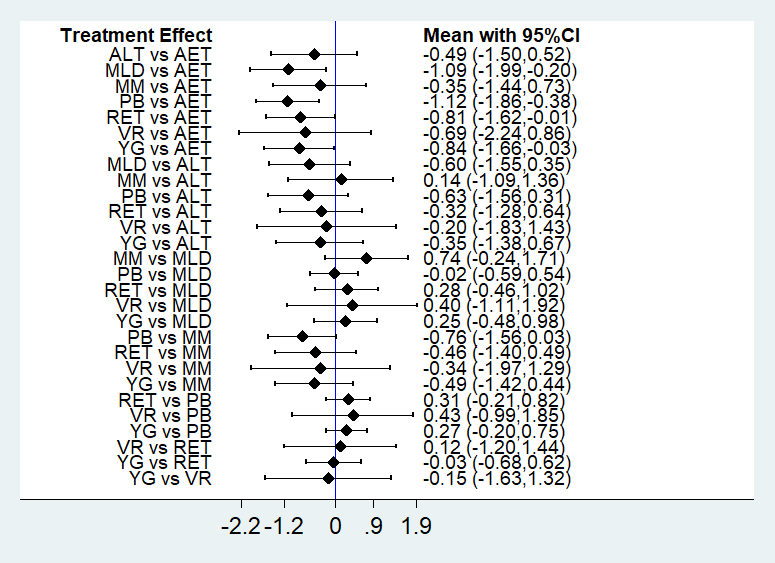


1. Grip strength **forest map**


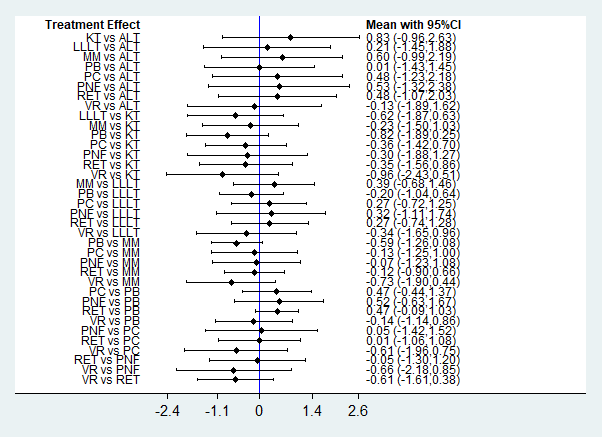


AE＝aquatic exercise，AET＝aerobic exercise，ALT＝aqua lymphatic therapy，ET＝electrotherapy，KT＝kinesio taping，LLLT＝low level laser therapy，MLD＝manual lymphatic drainage，MM＝mixed motion，MO＝moxibustion，PB＝placebo group，PC＝pneumatic circulation，PNF＝proprioceptive neuromuscular facilitation，RET＝resistance exercise，VR＝virtul reality，YG＝yoga，UG＝ultrasound therapy

**League tables**

**The following pages contain the league tables for each outcome (by individual physiotherapy type). Network meta-analyses for all the presented league tables were carried out using a random effects model.**

1. **Pain assessment League tables**

1. **Fatigue assessment League tables**

1. **Disabilities of Arm, Shoulder and Hand League tables**

1. **Quality of life (physical components) League tables**

1. **Quality of life (mental components) League tables**

1. Grip strength **League tables**

AE＝aquatic exercise，AET＝aerobic exercise，ALT＝aqua lymphatic therapy，ET＝electrotherapy，KT＝kinesio taping，LLLT＝low level laser therapy，MLD＝manual lymphatic drainage，MM＝mixed motion，MO＝moxibustion，PB＝placebo group，PC＝pneumatic circulation，PNF＝proprioceptive neuromuscular facilitation，RET＝resistance exercise，VR＝virtul reality，YG＝yoga，UG＝ultrasound therapy

**SUCRA rank**

1. **Pain assessment SUCRA rank**


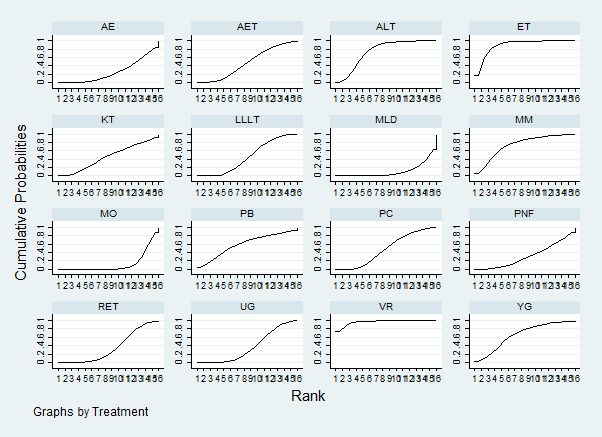


1. **Fatigue assessment SUCRA rank**


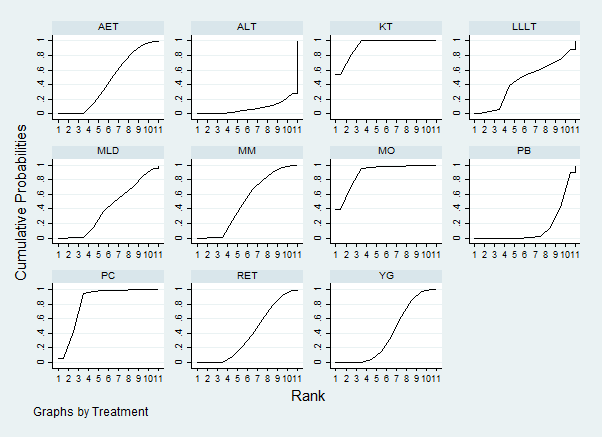


1. **Disabilities of Arm, Shoulder and Hand SUCRA rank**


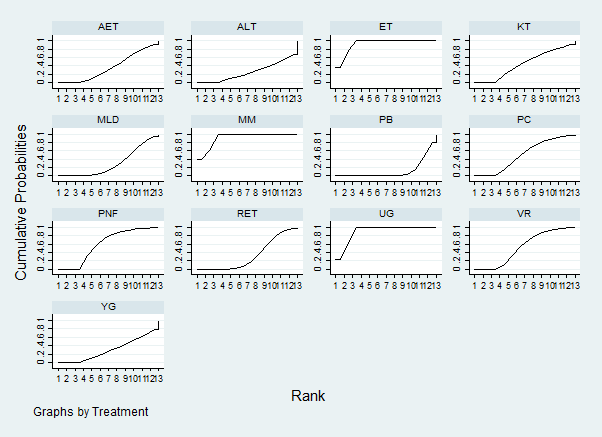


1. **Quality of life (physical components) SUCRA rank**


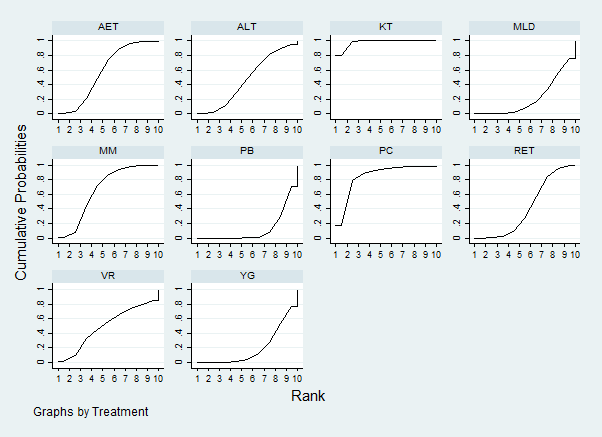


1. **Quality of life (mental components) SUCRA rank**


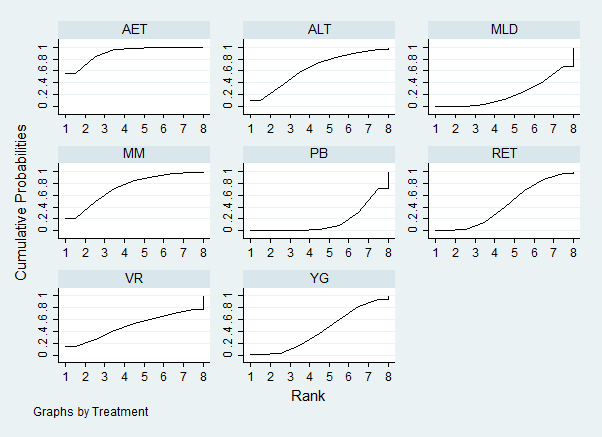


1. Grip strength **SUCRA rank**


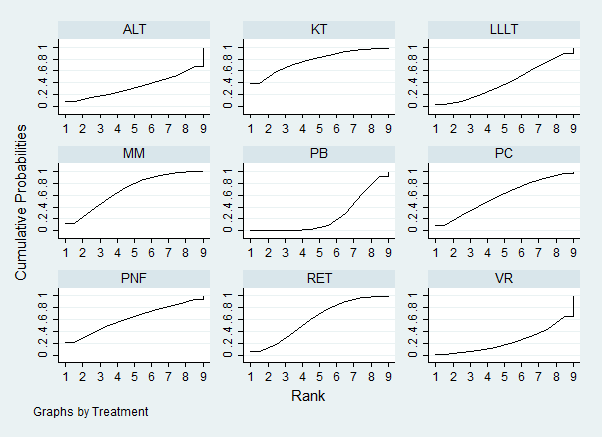


AE＝aquatic exercise，AET＝aerobic exercise，ALT＝aqua lymphatic therapy，ET＝electrotherapy，KT＝kinesio taping，LLLT＝low level laser therapy，MLD＝manual lymphatic drainage，MM＝mixed motion，MO＝moxibustion，PB＝placebo group，PC＝pneumatic circulation，PNF＝proprioceptive neuromuscular facilitation，RET＝resistance exercise，VR＝virtul reality，YG＝yoga，UG＝ultrasound therapy，**SUCRA**＝Surface Under The Cumulative Ranking Curve

**CINeMA (certainty of evidence)**

**CINeMA quality assessment of the comparisons in the network**

**We evaluated the certainty of evidence using the Confidence in Network Meta-Analysis (CINeMA) framework. CINeMA is a software, freely available as an open-source web application (https://cinema.ispm.unibe.ch/), which uses the R-package ‘meta’ and ‘netmeta’ to perform network meta-analysis of the data.**

**1. Within-study bias: We graded the risk of bias for the individual studies using the Cochrane Risk of Bias Version 2 (RoB2) assessment tool.RoB2 is structured into five domains that focuses on different aspects of trial design, conduct, and reporting. Based on the risk of bias for each domain, each study can be judged to have an overall ‘Low risk’ or ‘High’ risk of bias, or ‘Some concerns’can be expressed.**

**CINeMA combines the percentual per-study contribution for each judgement of risk of bias (according to RoB2) to evaluate within-study bias for each estimate from a network meta-analysis.Using CINeMA, we summarized RoB2 assessments for each pairwise comparison using the “Average RoB” rule, which applies a weighted average score for each relative effect estimate according to the percentage contribution of studies at each bias level.Based on these scores, estimates from each pairwise comparison was assigned a within-study bias of “No concerns”, “Some concerns”, or “Major concerns”.**

**2. Reporting bias: We used the Risk Of Bias due to Missing Evidence in Network meta-analysis (ROB-MEN) tool to assess reporting bias. ROB-MEN is a freely available open-source web application (https://cinema.ispm.unibe.ch/rob-men/). We assessed possible bias due to (i) thepresence of identified studies with unavailable results (within-study assessment of bias), and (ii)the potential for unpublished studies (across-study assessment of bias).**

**We assessed within-study assessment of bias by answering the following signaling question: “Was there any eligible study for which results for the outcome of interest were unavailable, likely because of the p-value, magnitude or direction of the result generated?”. If ‘yes’ could be answered to this question, we also addressed the following question: “Was the amount of information omitted from the synthesis sufficient to have a notable effect on the magnitude of the synthesized result?”. If both questions could be answered with a ‘yes’, within-study bias (selective outcome**

**reporting) was suspected for a given study.**

**Across-study assessment of bias for each pairwise comparison combines the contribution of direct comparisons to the network meta-analysis estimates with qualitative judgements of the risk of publication bias, and quantitative methods, including assessment of contour-enhanced funnel plots (where comparisons included at least 10 studies), meta-regression (using the variance as covariate), and statistical testing for small-study effects.**

**Based on the within-study and across-study assessments of bias, we assigned for all network meta-analysis estimates an overall ‘Low risk of bias’, ‘Some concerns’, or ‘High risk of bias’.**

**3. Indirectness: For the indirectness domain, similar to “Within‐study bias,” the**

**summary shows how many studies have been characterized as of low, moderate, and high indirectness at the top of the page. Subsequently, a bar graph shows the contribution of studies at each indirectness level to each NMA estimate. As for the “Within‐study bias” domain,users can select between “Majority,”“Average,” and “Highest” rules to summarize indirectness for each relative effect estimate. Areas are colored accordingly, while judgments under each rule are shown in the boxes. Manual changes can be made, and “Reset” and “Proceed”(to the “Imprecision” domain) buttons appear.**

**4.Imprecision: If the 95% confidence intervals (CIs) extended beyond the area of equivalence on the opposite side of the no effect line as the point estimate, so that the estimated treatment effect was compatible with clinically meaningful effects in both directions. We rated ‘no concerns’ if the CIs were entirely on one side of the no effect line, or if it was entirely within the area of equivalence.We rated ‘some concerns’, if the CIs extended into but not beyond the area of equivalence on the opposite side of the no effect line. Finally, we rated ‘major concerns’ if CIs extended beyond the area of equivalence on the opposite side of the no effect line.**

**5.Heterogeneity: Using CINeMA, we assessed the CIs and prediction intervals for each pairwise network estimate and checked whether the prediction intervals included values that would change the conclusions based on the CIs. When CIs and prediction intervals led to the same conclusions,we rated ‘no concerns’ in terms of heterogeneity.25 When CIs and prediction intervals led to conclusions that were somewhat different but of lesser impact for decision-making, we rated ‘some concerns’ in terms of heterogeneity.**

**6.Incoherence: When the assumption of transitivity holds, direct and indirect evidence should be in agreement and coherent. For estimates where both direct and indirect evidence were available,we used CINeMA to compare direct and indirect evidence, and downgraded comparisons that were significantly different at local node splitting test. If only direct or indirect evidence was available, CINeMA used a global design-by-treatment interaction test, and rates ‘no concerns’ where the p-value was >0.10, ‘some concerns’ if it was between 0.05 and 0.10, and ‘major concerns’ if it was <0.05.**

**Finally, we assigned each comparison an overall qualitative judgment of high, moderate, low, or very low certainty of evidence based on the level of concerns for the six CINeMA domains.25 We downgraded the overall certainty of evidence by one level for each domain with “some concerns” and two levels for each domain with “major concerns”, keeping in mind the fact that the six CINeMA domains are interconnected.We considered the domains jointly to avoid downgrading the overall level of confidence more than necessary for related concerns. An example to illustrate the interdependent nature of the six domains could be that indirectness relates to intransitivity, which can present as incoherence. Incoherence could be concealed by heterogeneity, which in turn could increase imprecision, thereby lowering the confidence for some comparisons. Heterogeneity might also be related to variability in within-study bias and/or the presence of reporting bias.**

1. **CINeMA (certainty of evidence)---Pain assessment**

1. **CINeMA (certainty of evidence)---Fatigue assessment**

1. **CINeMA (certainty of evidence)---Disabilities of Arm, Shoulder and Hand**

1. **CINeMA (certainty of evidence)---Quality of life (physical components)**

1. **CINeMA (certainty of evidence)---**Grip strength

**CINeMA＝Confidence In Network Meta-Analysis，**AE＝aquatic exercise，AET＝aerobic exercise，ALT＝aqua lymphatic therapy，ET＝electrotherapy，KT＝kinesio taping，LLLT＝low level laser therapy，MLD＝manual lymphatic drainage，MM＝mixed motion，MO＝moxibustion，PC＝pneumatic circulation，PNF＝proprioceptive neuromuscular facilitation，RET＝resistance exercise，VR＝virtul reality，YG＝yoga，UG＝ultrasound therapy

**Evaluation of inconsistency**

**We used stata17.0 software to test the inconsistency of the data. On the whole, no significant inconsistency was detected for all the result indicators that formed network evidence, which proved that the network analysis had good internal consistency.**

1. **Pain assessment**

**Side Direct Indirect Difference tau**

**Coef. Std. Err. Coef. Std. Err. Coef. Std. Err. P>|z|**

**A J * 1.018799 .8623847 .4364249 4.664743 .5823739 4.743789 0.902 .7745002**

**B C -.0009414 .8018858 -.8299894 .7670945 .829048 1.109703 0.455 .7834207**

**B J .2618471 .8177509 .1602935 .5667864 .1015536 .9950774 0.919 .7919769**

**B M -.4220863 .5687207 .2879381 .844448 -.7100244 1.01828 0.486 .7822275**

**B P -.2678732 .8529269 -.1980073 .6035269 -.069866 1.044859 0.947 .7913381**

**C G -.7006147 .8178068 .4283943 .5948 -1.129009 1.011235 0.264 .7705458**

**C H 2.423318 .7859493 -.0742153 .5419869 2.497533 .9547073 0.009 .6876294**

**C J .7657335 .9316534 .5872239 .5049527 .1785095 1.059698 0.866 .7879582**

**C M -.5850824 .7826879 .6917688 .5885642 -1.276851 .9792173 0.192 .7634581**

**D G .5510436 .8718543 .833739 .5634406 -.2826955 1.038073 0.785 .792197**

**D J * 1.394536 .458588 1.112391 .9310179 .2821455 1.037601 0.786 .7921815**

**D N * -.2601027 .7344777 -11.58284 2.149072 11.32274 2.248731 0.000 .6201564**

**E J 3.370654 .8940157 1.119527 .7124854 2.251127 1.143197 0.049 .73358**

**E K .8118942 .5652118 3.062566 .9936721 -2.250672 1.143177 0.049 .7335794**

**F J .6340048 .9358073 .5418632 .9562205 .0921416 1.337946 0.945 .7894555**

**F K -.0849852 .8417091 .008125 1.040111 -.0931102 1.338022 0.945 .7894579**

**G J .4292886 .3441474 1.219676 .6878672 -.7903878 .7690682 0.304 .7816103**

**H J -.0374907 .4738545 -.2312301 .5425106 .1937394 .7202262 0.788 .7898839**

**H L .0979558 .8025569 -.9040343 .7869218 1.00199 1.124063 0.373 .7774697**

**H M -.0480663 .8183289 -.679691 .4864176 .6316247 .9519792 0.507 .7841177**

**H P .1640005 .8979489 -.7192012 .4591255 .8832017 1.008518 0.381 .7780946**

**I K * .7700787 .5944112 .7403694 44.77228 .0297093 44.77621 0.999 .7740443**

**J K -.1744742 .5855301 -1.120991 .6010136 .9465164 .8391136 0.259 .772396**

**J L * -.4346087 .5837011 .2835373 1.171551 -.718146 1.308617 0.583 .7858262**

**J M -.4056885 .4245886 -.3782086 .4795959 -.0274799 .6405772 0.966 .7914349**

**J N * -6.599343 1.09237 4.72515 1.63031 -11.32449 2.24884 0.000 .6201564**

**J O -.6333062 .8525064 -1.616407 .8906215 .9831008 1.232873 0.425 .780318**

**J P -.4754794 .2776385 -.016581 .7108202 -.4588984 .7631004 0.548 .7849373**

**K M .0384243 .8358148 .3459489 .5907607 -.3075245 1.023516 0.764 .7901871**

**L M .1515667 .8445581 -.3003901 .7483282 .4519569 1.128222 0.689 .7887829**

**M O -1.154437 .8292327 -.1717717 .9124045 -.9826655 1.232927 0.425 .7803206**

A＝aquatic exercise，B＝aerobic exercise，C＝aqua lymphatic therapy，D＝electrotherapy，E＝kinesio taping，F＝low level laser therapy，G＝manual lymphatic drainage，H＝mixed motion，I＝moxibustion，J＝placebo group，K＝pneumatic circulation，L＝proprioceptive neuromuscular facilitation，M＝resistance exercise，N＝ultrasound therapy，O＝virtul reality，P＝yoga

1. **Fatigue assessment**

**Side Direct Indirect Difference tau**

**Coef. Std. Err. Coef. Std. Err. Coef. Std. Err. P>|z|**

**A H .4859987 .3373492 .2951269 .4421937 .1908718 .5556856 0.731 .6138822**

**A J -.0194502 .4092722 .2270908 .4766509 -.2465411 .6282256 0.695 .61327**

**A K .1257818 .3823267 .0082015 .4142689 .1175803 .5631294 0.835 .614879**

**B H * -.6053182 .6574865 .8610079 63.25781 -1.466326 63.26128 0.982 .6027429**

**C H * 3.24735 .7828608 .7346892 21.09211 2.512661 21.10663 0.905 .6027597**

**C I * .5342893 .6586082 3.544881 28.30777 -3.010592 28.31542 0.915 .6027586**

**D E * .1379056 .7020192 .0585268 63.26505 .0793788 63.26888 0.999 .6027455**

**E H * .3884737 .4892569 .5059236 25.82724 -.1174499 25.8319 0.996 .6027479**

**F H .7142917 .2941788 -.8865941 .7785743 1.600886 .832274 0.054 .5812242**

**F K -1.175941 .7636282 .4264241 .3317868 -1.602365 .8325927 0.054 .5812307**

**G I * .3633458 .6694554 -4.590336 63.29436 4.953681 63.2979 0.938 .6027529**

**H J -.306088 .2673327 -.5138765 .7275484 .2077885 .7747622 0.789 .6135392**

**H K -.2848362 .1618847 -1.032118 .555863 .7472816 .5789872 0.197 .602422**

**A＝**aerobic exercise，B＝aqua lymphatic therapy，C＝kinesio taping，D＝low level laser therapy，E＝manual lymphatic drainage，F＝mixed motion，G＝moxibustion，H＝placebo group，I＝pneumatic circulation，J＝resistance exercise，K＝yoga

1. **Disabilities of Arm, Shoulder and Hand**

**Side Direct Indirect Difference tau**

**Coef. Std. Err. Coef. Std. Err. Coef. Std. Err. P>|z|**

**A G .3832915 .791687 .6945872 .8717414 -.3112956 1.177583 0.792 .7350365**

**A J .2151833 .7991443 -.0951625 .866371 .3103458 1.178656 0.792 .735113**

**B G * .1108721 .8304706 1.032627 63.26664 -.9217553 63.27249 0.988 .6813355**

**C G * 4.687833 .9738869 -.3874994 63.26822 5.075333 63.27731 0.936 .6813309**

**C K . . . . . . . .**

**D G * .7612804 .7648156 1.087065 63.26805 -.325785 63.27257 0.996 .6813348**

**E G * .3537227 .318925 1.03946 25.84814 -.6857371 25.85006 0.979 .6814106**

**F G * 4.545081 .7827189 1.043614 63.26617 3.501466 63.27101 0.956 .681332**

**G H -1.117387 .7669196 -.5712745 .8550822 -.5461129 1.148621 0.634 .7257911**

**G I -1.492741 .7866141 -.7555613 .8601844 -.7371798 1.165624 0.527 .7136961**

**G J -.5910909 .4024158 -.1033292 .6323121 -.4877617 .7496531 0.515 .7183648**

**G K * -4.48987 .9597603 .5854679 63.2723 -5.075338 63.28131 0.936 .6813308**

**G L -.1198234 .7074305 -1.494486 .6093426 1.374663 .9336794 0.141 .6235168**

**G M * -.3354422 .7643275 -1.064615 63.26646 .7291733 63.27096 0.991 .6813354**

**H J .1739547 .7754071 .7200361 .8474008 -.5460814 1.148627 0.634 .7257914**

**I J .7416896 .8008643 .6618901 .8840497 .0797995 1.189821 0.947 .7388536**

**I L -1.29e-11 .7992665 .5535374 .8858036 -.5535374 1.193095 0.643 .7264405**

**J L -.9673143 .7442576 -.0450689 .672671 -.9222454 1.003198 0.358 .6919433**

**A＝**aerobic exercise，B＝aqua lymphatic therapy，C＝electrotherapy，D＝kinesio taping，E＝manual lymphatic drainage，F＝mixed motion，G＝placebo group，H＝pneumatic circulation，I＝proprioceptive neuromuscular facilitation，J＝resistance exercise，K＝ultrasound therapy，L＝virtul reality，M＝yoga

1. **Quality of life (physical components)**

**Side Direct Indirect Difference tau**

**Coef. Std. Err. Coef. Std. Err. Coef. Std. Err. P>|z|**

**A B .0455266 .5067479 -.2981127 .4905464 .3436393 .7048946 0.626 .4769895**

**A F -.1792935 .2918429 -.9471954 .3273612 .7679019 .4381978 0.080 .4383705**

**A H -.1118934 .2719037 -.4357198 .4067996 .3238264 .4892396 0.508 .4740311**

**A J -1.759931 .4253762 .0248835 .2579509 -1.784814 .4979614 0.000 .3452963**

**B D -.4570293 .5481574 -.2192891 .4366943 -.2377403 .7008413 0.734 .4776061**

**B E .1718211 .6101769 .3353542 .4418067 -.1635332 .7533319 0.828 .4783555**

**B F .0882724 .4904072 -.6776162 .3801319 .7658886 .6198569 0.217 .4597888**

**B H .0135938 .5095968 -.1506844 .4414444 .1642782 .6735684 0.807 .480411**

**C F * -1.987847 .4512219 -.8596167 25.82497 -1.12823 25.82888 0.965 .4670886**

**C G * -.4957131 .5368889 -2.93803 63.2542 2.442317 63.25646 0.969 .4670857**

**D F -.1096138 .243224 .1273495 .6568782 -.2369632 .7006632 0.735 .4776017**

**E F -.6924418 .2692526 -.5292833 .7034558 -.1631585 .7530952 0.828 .4783474**

**F H .2460496 .2031678 .5296243 .368966 -.2835747 .4212052 0.501 .4729136**

**F J .0951525 .1930859 -.2360837 .549399 .3312363 .581271 0.569 .4741903**

**H I * .1622993 .5339172 .4229111 63.25036 -.2606118 63.25261 0.997 .4670787**

**H J -.1929656 .5895321 -.2647044 .2617866 .0717388 .645043 0.911 .4783892**

A＝aerobic exercise，B＝aqua lymphatic therapy，C＝kinesio taping，D＝manual lymphatic drainage，E＝mixed motion，F＝placebo group，G＝pneumatic circulation，H＝resistance exercise，I＝virtul reality，J＝yoga

1. **Quality of life (mental components)**

**Side Direct Indirect Difference tau**

**Coef. Std. Err. Coef. Std. Err. Coef. Std. Err. P>|z|**

**A B -.2434093 .6582966 -.9131323 .8617449 .669723 1.084182 0.537 .6355593**

**A E -1.070718 .5058238 -1.182652 .5977861 .1119337 .7795119 0.886 .6451436**

**A F .1074477 .5846407 -1.448041 .4903948 1.555488 .7630709 0.042 .560691**

**A H -2.734129 .4475894 .231368 .3393291 -2.965497 .559553 0.000 .3479995**

**B C -.9225432 .6942968 -.279518 .700308 -.6430252 .9861438 0.514 .6352433**

**B F .3542517 .6096239 -1.221306 .7061383 1.575558 .9325159 0.091 .585186**

**C E -.0856283 .3079783 .5538237 .9363769 -.639452 .9857256 0.517 .6352273**

**D E * -.7627159 .4054461 -2.264435 36.52887 1.501719 36.53109 0.967 .6241572**

**E F .2598382 .3116617 .4368675 .5331687 -.1770292 .6179168 0.775 .6397549**

**E H .2695474 .2638846 .3084726 .7273776 -.0389252 .7730664 0.960 .6428701**

**F G * .120032 .6755482 1.622743 63.26087 -1.502711 63.26448 0.981 .6241541**

**F H 1.044328 .6962149 -.3174642 .3551115 1.361793 .7815494 0.081 .5899334**

**A＝**aerobic exercise，B＝aqua lymphatic therapy，C＝manual lymphatic drainage，D＝mixed motion，E＝placebo group，F＝resistance exercise，G＝virtul reality，H＝yoga

1. Grip strength

Side Direct Indirect Difference tau

Coef. Std. Err. Coef. Std. Err. Coef. Std. Err. P>|z|

A E * .0136074 .7389696 -.2713056 7.67173 .284913 7.707238 0.971 .6211403

B E .2918822 .4886326 -2.51002 .5773093 2.801902 .7563378 0.000 .3548857

B F -1.411451 .4605643 1.391094 .5999858 -2.802545 .7563746 0.000 .3548855

C E -.1691497 .5110732 -.2952837 .9276919 .126134 1.059125 0.905 .6585399

C F .2048077 .7206581 .3313333 .7766303 -.1265255 1.059482 0.905 .6585643

D E * -.6915302 .3999151 -.2273219 .7591738 -.4642083 .8580272 0.588 .6480442

D G * -.0708038 .6920224 -.0737611 1.464027 .0029573 1.61974 0.999 .6627753

D H .1793453 .6887034 -.2845133 .512135 .4638586 .858251 0.589 .6480637

E F 1.540315 .5127924 -.4917521 .494911 2.032067 .712666 0.004 .440086

E G * .5192298 .6900178 .5217184 1.466672 -.0024886 1.619573 0.999 .6627674

E H .3029935 .3234771 1.040169 .5964884 -.7371751 .6784126 0.277 .6120949

E I .2997484 .7108898 -.6298425 .748283 .9295909 1.032131 0.368 .6264758

H I -1.020823 .6845233 -.091232 .7725306 -.9295907 1.03217 0.368 .6264787

A＝aqua lymphatic therapy，B＝kinesio taping，C＝low level laser therapy，D＝mixed motion，E＝placebo group，F＝pneumatic circulation，G＝proprioceptive neuromuscular facilitation，H＝resistance exercise，I＝virtul reality

P-value (P>|z|): The significance level for testing whether the "difference is zero". P > 0.05: This indicates that there is no statistically significant difference between the direct comparison and the indirect comparison, meaning the two are consistent.

**Publication bias**

1. **Pain assessment funnel diagram**


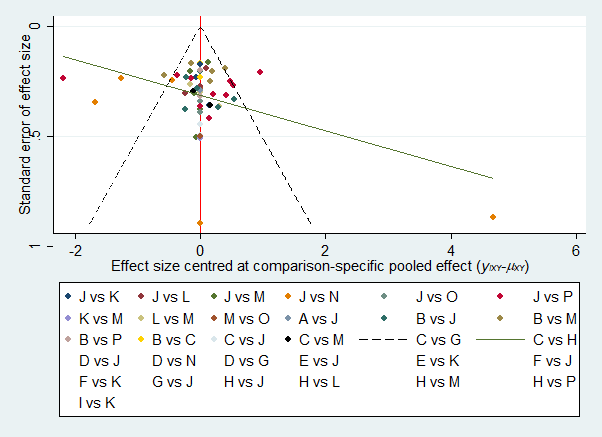


A＝aquatic exercise，B＝aerobic exercise，C＝aqua lymphatic therapy，D＝electrotherapy，E＝kinesio taping，F＝low level laser therapy，G＝manual lymphatic drainage，H＝mixed motion，I＝moxibustion，J＝placebo group，K＝pneumatic circulation，L＝proprioceptive neuromuscular facilitation，M＝resistance exercise，N＝ultrasound therapy，O＝virtul reality，P＝yoga

1. **Fatigue evaluation funnel diagram**


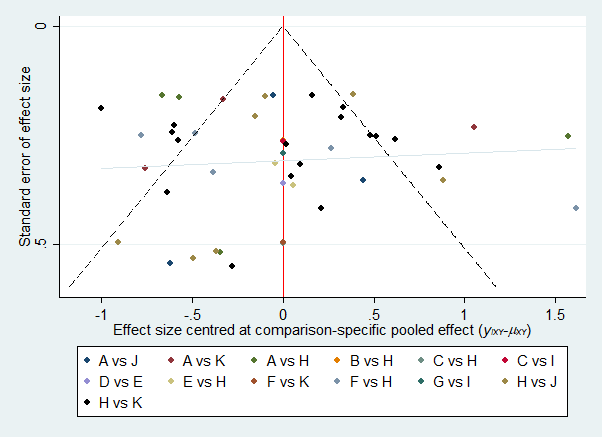


**A＝**aerobic exercise，B＝aqua lymphatic therapy，C＝kinesio taping，D＝low level laser therapy，E＝manual lymphatic drainage，F＝mixed motion，G＝moxibustion，H＝placebo group，I＝pneumatic circulation，J＝resistance exercise，K＝yoga

1. **Disabilities of Arm, Shoulder and Hand funnel diagram**


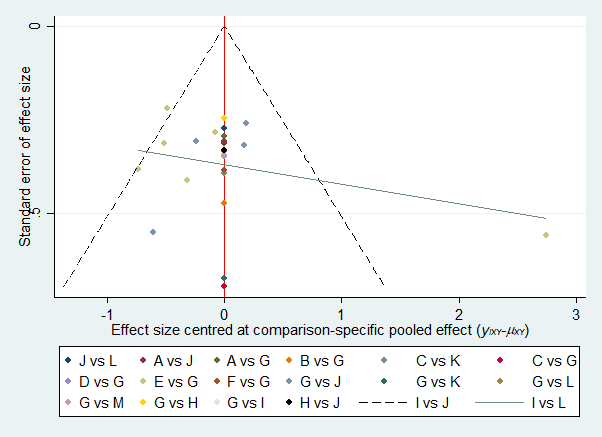


**A＝**aerobic exercise，B＝aqua lymphatic therapy，C＝electrotherapy，D＝kinesio taping，E＝manual lymphatic drainage，F＝mixed motion，G＝placebo group，H＝pneumatic circulation，I＝proprioceptive neuromuscular facilitation，J＝resistance exercise，K＝ultrasound therapy，L＝virtul reality，M＝yoga

1. **Quality of life (physical components) funnel diagram**


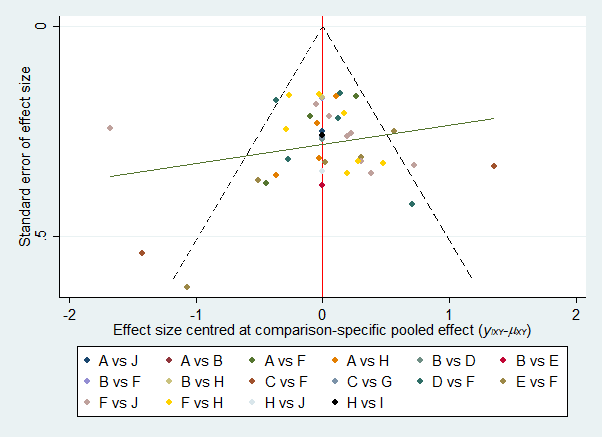


A＝aerobic exercise，B＝aqua lymphatic therapy，C＝kinesio taping，D＝manual lymphatic drainage，E＝mixed motion，F＝placebo group，G＝pneumatic circulation，H＝resistance exercise，I＝virtul reality，J＝yoga

1. **Quality of life (mental components) funnel diagram**


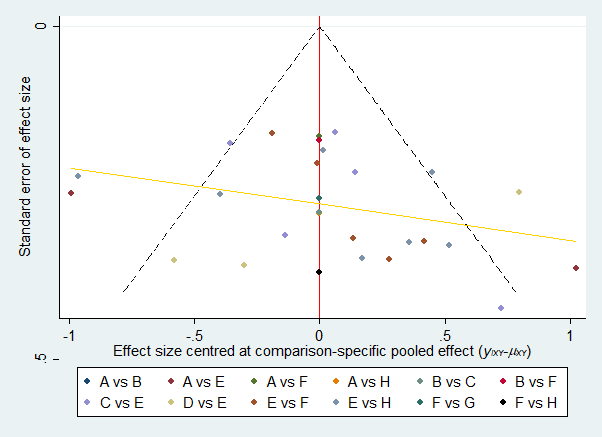


**A＝**aerobic exercise，B＝aqua lymphatic therapy，C＝manual lymphatic drainage，D＝mixed motion，E＝placebo group，F＝resistance exercise，G＝virtul reality，H＝yoga

1. Grip strength **funnel diagram**


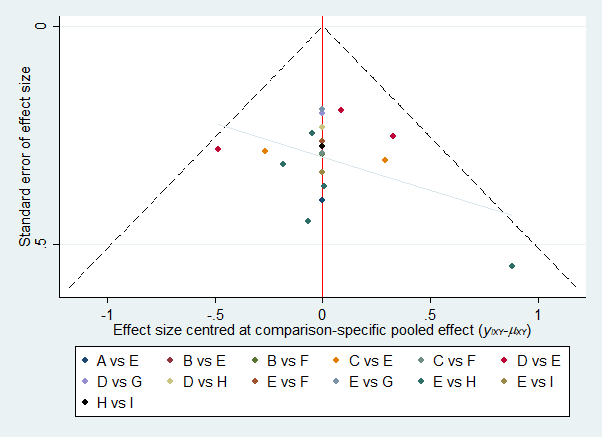


A＝aqua lymphatic therapy，B＝kinesio taping，C＝low level laser therapy，D＝mixed motion，E＝placebo group，F＝pneumatic circulation，G＝proprioceptive neuromuscular facilitation，H＝resistance exercise，I＝virtul reality
